# The tsRNAs (tRFdb-3013a/b) serve as novel biomarkers for colon adenocarcinomas

**DOI:** 10.18632/aging.205590

**Published:** 2024-03-06

**Authors:** Lihong Tan, Xiaoling Wu, Zhurong Tang, Huan Chen, Weiguo Cao, Chunjie Wen, Guojun Zou, Hecun Zou

**Affiliations:** 1 Chongqing Medical and Pharmaceutical College, Chongqing 401331, China; 2 Yueyang Hospital of Traditional Chinese Medicine, Yueyang 414000, China; 3 Chongqing Medical University, Chongqing 400016, China

**Keywords:** tsRNA, tRFdb-3013a, tRFdb-3013b, colon adenocarcinomas, ST3GAL1

## Abstract

The tsRNAs (tRNA-derived small RNAs) are a novel class of small non-coding RNAs derived from transfer-RNAs. Colon adenocarcinoma (COAD) is the most malignant intestinal tumor. This study focused on the identification and characterization of tsRNA biomarkers in colon adenocarcinomas. Data processing and bioinformatic analyses were performed with the packages of R and Python software. The cell proliferation, migration and invasion abilities were determined by CCK-8 and transwell assays. Luciferase reporter assay was used to test the binding of tsRNA with its target genes. With computational methods, we identified the tRNA fragments profiles within COAD datasets, and discriminated forty-two differentially expressed tsRNAs between paired colon adenocarcinomas and non-tumor controls. Among the fragments derived from the 3′ end of tRNA-His-GUG (a histidyl-transfer-RNA), tRFdb-3013a and tRFdb-3013b (tRFdb-3013a/b) were notably decreased in colon and rectum adenocarcinomas, especially, tRFdb-3013a/b might tend to be down-regulated in patients with lymphatic or vascular invasion present. The clinical survival of colorectal adenocarcinoma patients with low tRFdb-3013a/b expression was significantly worse than that of high expression patients. In colon adenocarcinoma cells, tRFdb-3013a could have inhibited cell proliferations, and reduced cell migration and invasion abilities. The enrichment analyses showed that most of tRFdb-3013a correlated-genes were enriched in the extracellular matrix associated GO terms, phagosome pathway, and a GSEA molecular signature pathway. Additionally, the 3′UTR of ST3GAL1 mRNA was predicted to contain the binding site of tRFdb-3013a/b, tRFdb-3013a/b might directly target and regulate ST3GAL1 expression in colon adenocarcinomas. These results suggested that tRFdb-3013a/b might serve as novel biomarkers for diagnosis and prognosis of colon adenocarcinomas, and act a key player in the progression of colon adenocarcinomas.

## INTRODUCTION

The tsRNAs (tRNA-derived small RNAs) are a novel class of small non-coding RNA derived from transfer-RNA (tRNA), and also called as tRNA-derived fragments (tRFs) [1]. The tRNA fragments are produced through cleavage of mature or pre-mature tRNAs at various positions, the fragments are short and range in length from 16 to 35 nucleotides [2]. A general classification of tsRNAs is based on their position in relation to the parental tRNA sequence, which can be classified into some distinct categories, including 5′-tRFs, 3′-tRFs, and internal tRFs, these tsRNAs originate precisely from the span of mature tRNAs [2, 3]. Two remaining tsRNAs: 5′U-tRFs, which derived from the 5′-leader sequence of primary tRNA genes; and tRF-1 that comprise part of the 3′-trailer sequence [2]. Numerous researches suggested that the generation of tRNAs into specific small fragments was a regimented process, did not result from degradation [4]. These cleavages of tRNAs are context-dependent, including sex, ethnicity, disease, and tissue type of individuals. These dependencies increase the urgency of understanding the regulatory roles of tsRNAs, such efforts are gaining momentum, and comprise experimental and computational approaches [5, 6].

These efforts have discriminated more than thousands of microRNA isoforms via analysis of datasets in The Cancer Genome Atlas (TCGA) [7, 8]. It is worth mentioning that tsRNA is sequenced at an abundance comparable to that of the abundant miRNA [9]. Several studies have linked the tsRNAs to different human cancers, including glioma [10], liver [11] and breast cancer, etc. [12]. Growing evidences demonstrated that many tsRNAs were differentially expressed in the tissues of multiple tumor patients, these dysregulated tRNA fragments might contribute to various biology processes of cancer development and progression [13]. Colon adenocarcinoma (COAD) is the most common type of primary tumor originating in the intestinal tract [14], colon adenocarcinomas may be the higher mortality among gastrointestinal tumors based on epidemiological statistical reports [15]. The pathogenesis of colon adenocarcinomas is extremely complicated, as it involves the aberrant activation of proto-oncogenes and inactivation of anti-oncogenes [14, 16]. However, it is not clear whether the tsRNAs would serve as novel molecular biomarkers for diagnosis, prognosis and target therapy of colon adenocarcinomas.

In the present study, we aimed to identify the tsRNAs profiles within the sncRNA-seq data of human colon adenocarcinomas samples via a deterministic and exhaustive tsRNAs mining pipeline, then investigate the expression patterns and biology function of tRFdb-3013a and tRFdb-3013b in colon adenocarcinomas. Additionally, the relationships between tRFdb-3013a/b and its target genes are also studied in order to reveal the underlying mechanisms.

## RESULTS

### The identifications of tsRNAs within colon adenocarcinomas data

Firstly, we collected more than thousands of tsRNA signatures from tRFdb [9] and tRFexplorer databases, the tsRNA sequences have mapped within the specific regions of tRNA (transfer-RNA) as described previously [17]. As statistical analysis, these tsRNA fragments are mainly derived from 5′ end (5’-tRFs) and 3′ end of mature tRNA, as well as the 5′ leader (5′U-tRF) and 3′ trailer regions of primary tRNA-gene. With computational approaches, we determined two hundreds of tsRNAs and assessed their expression profiles within the sncRNA-seq data of TCGA-COAD, the summary of our identification pipeline was showed in Supplementary Figure 1A. After filtering, a total of 192 tsRNAs with available expression abundances were identified within COAD samples (Supplementary Table 3), of which about thirty-two percents were derived from the 3′ trailer regions (tRF-1s) of primary tRNA genes, and thirty percent tsRNAs from the 3′ end (3’-tRFs) of mature tRNAs (Supplementary Figure 1C). As for the chromosomes, more than thirty-five percent of tsRNAs are originated from the human chr1 region, and nearly twenty percent originated from the chr6 region (Supplementary Figure 1B).

**Figure 1:**
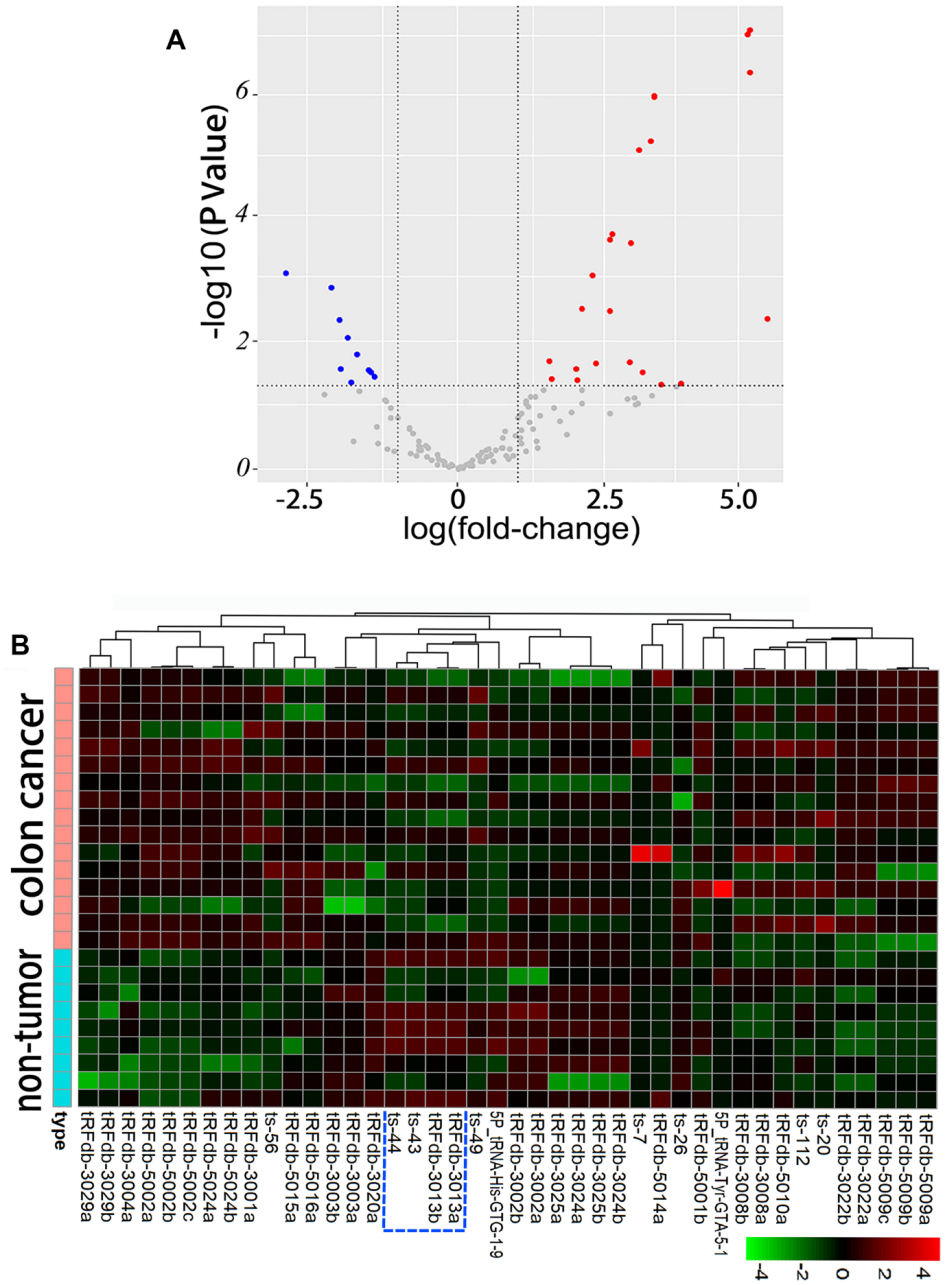
**Differential expression analysis between paired colon adenocarcinomas (n=16) and non-tumor controls (n=9) of TCGA-COAD dataset.** (**A**) Volcano plots of the differentially expressed tsRNAs, including 15 up-regulated and 27 down-regulated tsRNAs. (**B**) Hierarchical clustering and heatmap of differentially expressed tsRNAs, the up-regulated tsRNAs are clustered in red-shaded areas, and the green-shaded areas indicate down-regulation.

Based on the differential expression analysis between paired colon adenocarcinoma samples and non-tumor controls, we discriminated a total of forty-two differentially expressed tsRNA-genes (DEGs), of which fifteen were down-regulated and twenty-seven were up-regulated (Figure 1A and Supplementary Table 4) in paired colon adenocarcinomas compared to non-tumor controls. Hierarchical cluster analysis and heatmap of differentially expressed tsRNA genes were performed and exhibited in Figure 1B. The expression patterns of tsRNAs were significantly distinguished between paired colon adenocarcinomas and non-tumor groups. As shown in the blue dotted line frame of Figure 1B, several tsRNAs (including ts-43, ts-44, tRFdb-3013a and tRFdb-3013b) caught our attention that derived from tRNA-His-GUG, which is a histidyl-transfer-RNA [18].

### Significance of tRFdb-3013a and tRFdb-3013b within colon adenocarcinomas

**Figure 2:**
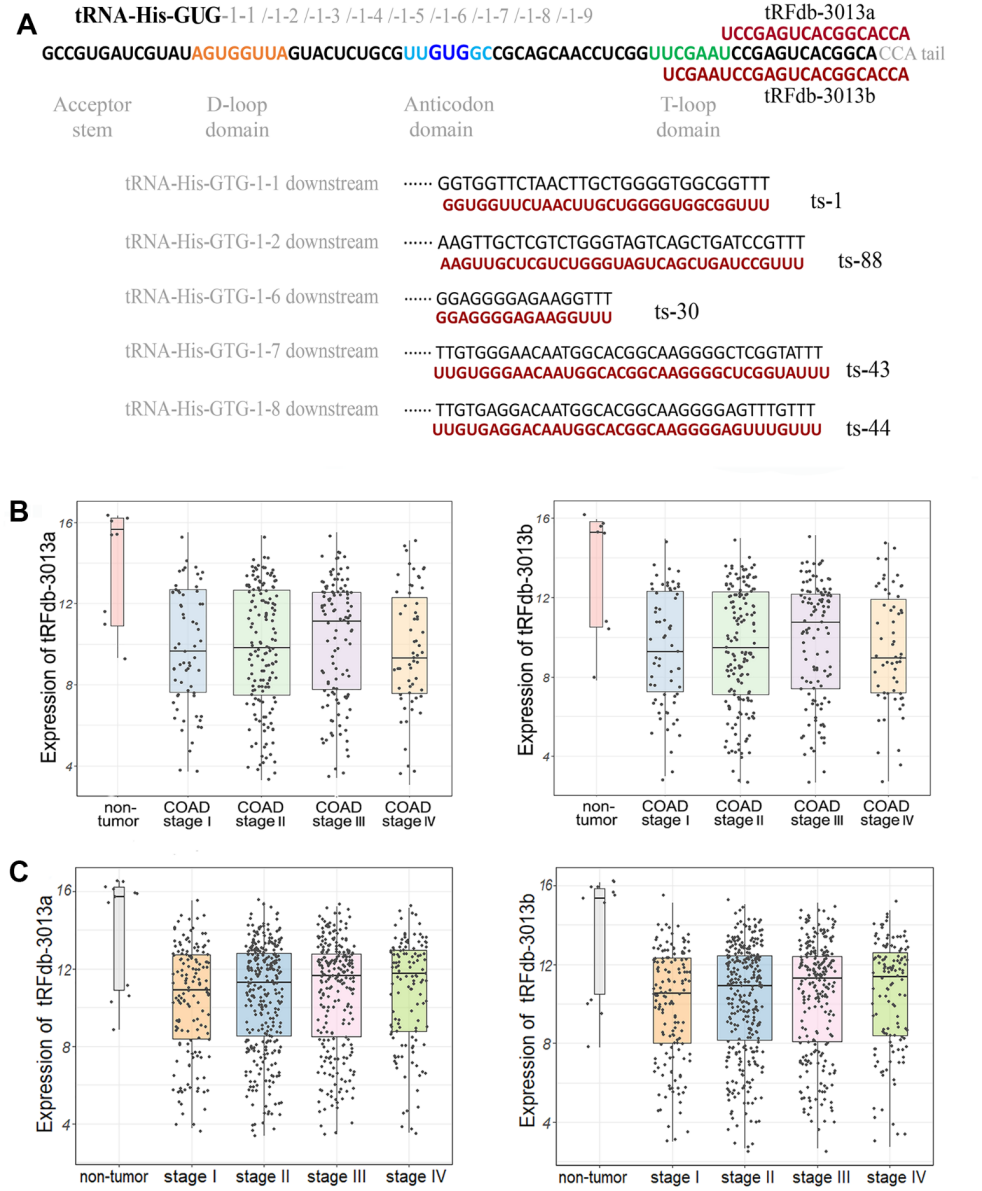
(**A**) Representative images of the sequence alignments between tsRNAs and its tRNA sources. (**B**) The scatter plus box plots of tRFdb-3013a and tRFdb-3013b expression (log_2_TPM) between non-tumor controls and colon adenocarcinomas (COAD) with four stages (both *P* <0.01). (**C**) The scatter plus box plots of tRFdb-3013a and tRFdb-3013b expression (log_2_TPM) between non-tumor controls and colon plus rectal adenocarcinomas (COAD-READ) with four stages (both *P* <0.01).

As shown in Figure 2A, we have identified seven tsRNA fragments derived from tRNA-His-GUG or *tRNA-His-GTG* gene in the sncRNA-seq datasets of colon adenocarcinomas, there are two 3’-tRFs fragments (tRFdb-3013a and tRFdb-3013b) that can re-map to the cleavages of 3′ end of several different mature tRNA-His-GUG isoforms (Supplementary Figure 2A), and five tRF-1 fragments (ts-30, ts-43, ts-44, ts-1 and ts-88) that can re-map to the down-stream regions (35 bases) of primary *tRNA-His-GTG* genes. To reveal the implication of these tsRNAs in colon adenocarcinomas, the expression patterns of above tsRNAs were analyzed in the samples of TCGA-COAD dataset. It showed that the expressions of four tsRNAs were down-regulated in colon adenocarcinoma samples compared to paired controls (Supplementary Figure 2B, all *P* <0.05, Supplementary Table 5), and compared with non-tumor controls, the expressions of tRFdb-3013a and tRFdb-3013b (known simply as tRFdb-3013a/b) were significantly down-regulated in colon adenocarcinoma and/or rectum adenocarcinoma samples with four stages (Figure 2B, 2C, all *P* <0.01). In our clinical tissue samples detections, it also demonstrated that the tRFdb-3013a/b expressions were down-regulated in colon adenocarcinoma compared to non-tumor controls (Supplementary Figure 3A, 3B).

**Figure 3:**
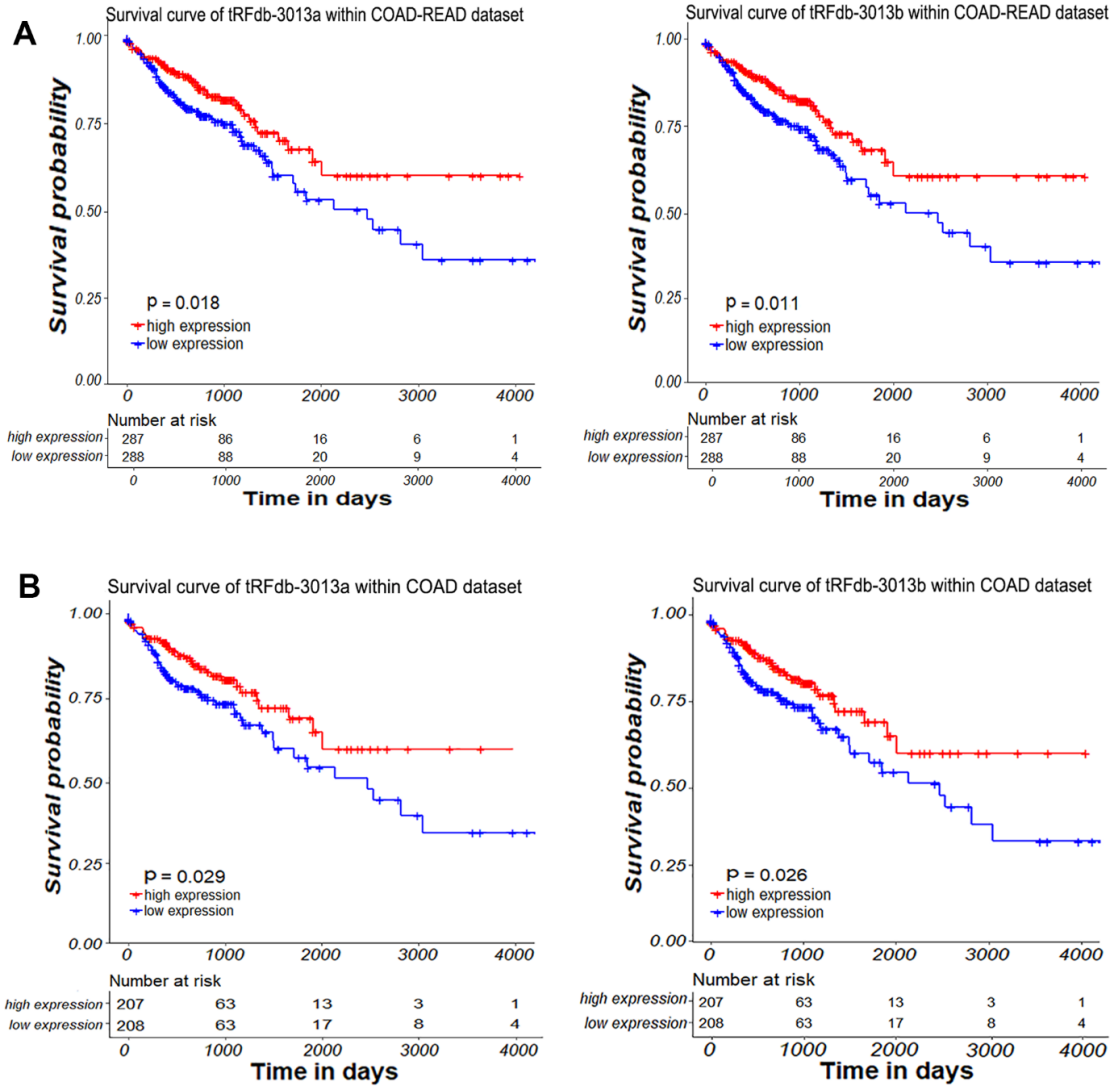
**The survival analysis of tRFdb-3013a and tRFdb-3013b.** (**A**) KM curve survival analysis with log-rank tests based on tRFdb-3013a and tRFdb-3013b expression within colon adenocarcinoma plus rectum adenocarcinoma (COAD-READ) samples (n=805) of TCGA datasets. (**B**) KM survival analysis with log-rank tests based on tRFdb-3013a and tRFdb-3013b expression within colon adenocarcinoma samples (n=587) of TCGA-COAD dataset.

**Figure 4:**
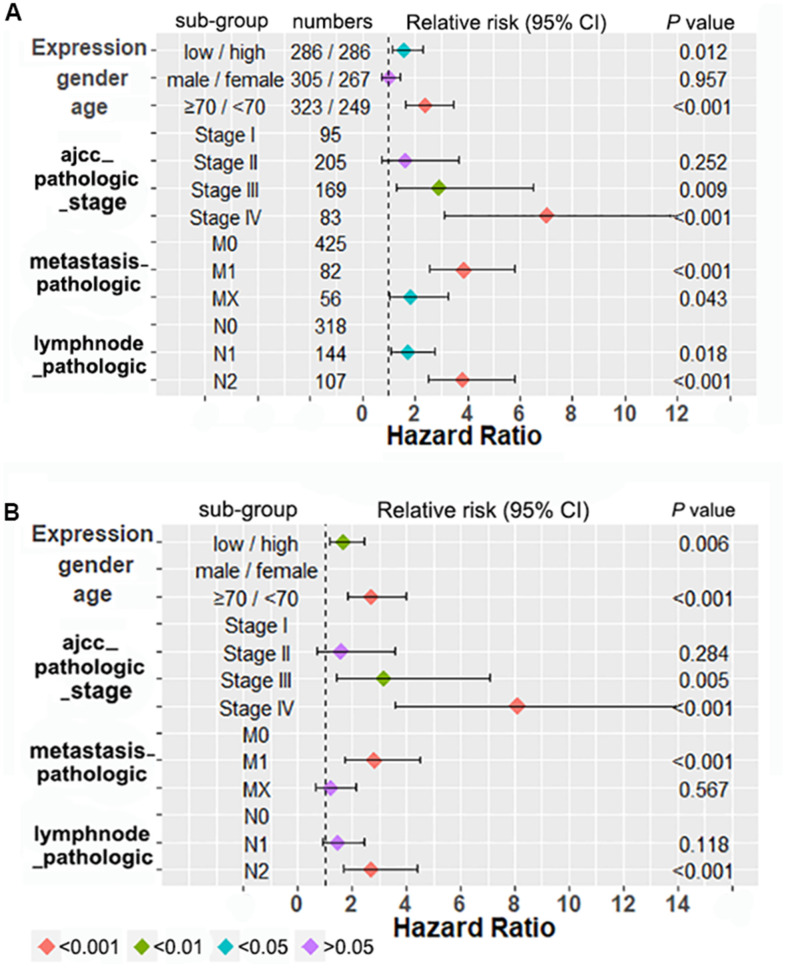
**Cox regression analysis of overall survival within colon adenocarcinoma plus rectum adenocarcinoma patients.** (**A**) Univariate analysis of tRFdb-3013a expressions and other risk factors within patients of TCGA dataset. (**B**) Multivariate Cox regression analysis of tRFdb-3013a expressions and other risk factors within patients.

Subsequently, we investigated the relationship between tsRNA expressions and overall survival of colon and/or rectum adenocarcinoma samples using KM (Kaplan–Meier) curve analysis [10]. Based on the median value of tsRNAs expression, primary tumor samples were divided into low-expressions and high-expressions group [19]. As shown in Figure 3B, the clinical survival of colon adenocarcinoma patients with low-expressions of tRFdb-3013a or tRFdb-3013b was worse than that of the high-expression patients (*P* =0.029 and *P* =0.026); As for colon adenocarcinoma plus rectum adenocarcinoma (COAD-READ), the clinical survival of patients with low-expressions of tRFdb-3013a or tRFdb-3013b were also significantly worse than that of the high-expression patients (*P* =0.018 and *P* =0.011, Figure 3A). What's more, the Cox regression analysis of overall survival within COAD-READ patients were performed [20]. As shown in Figure 4A, the forest plot described that univariate Cox regression analysis of patients showed that low tRFdb-3013a expression (*P* =0.012), increased age (*P* <0.001), high AJCC pathologic stage (*P* <0.01 for stage III and *P* <0.001 for stage IV), advanced metastasis pathologic spread (*P* <0.001 for M1) and lymph node pathologic spread (P <0.001 for N2) were the risk factors associated with prognosis. Subsequent multivariate Cox regression analysis results revealed that low tRFdb-3013a expression (HR=1.684, *P* = 0.006) were an independent prognosis factor for survival of patients (Figure 4B), in addition to the increased age, advanced metastasis and lymph node pathologic spread. Similar results were also obtained from the Cox regression analysis of tRFdb-3013b. These results suggested that down-regulated tRFdb-3013a/b was associated with poor survival prognosis of colon adenocarcinoma and rectum adenocarcinoma patients.

**Figure 5:**
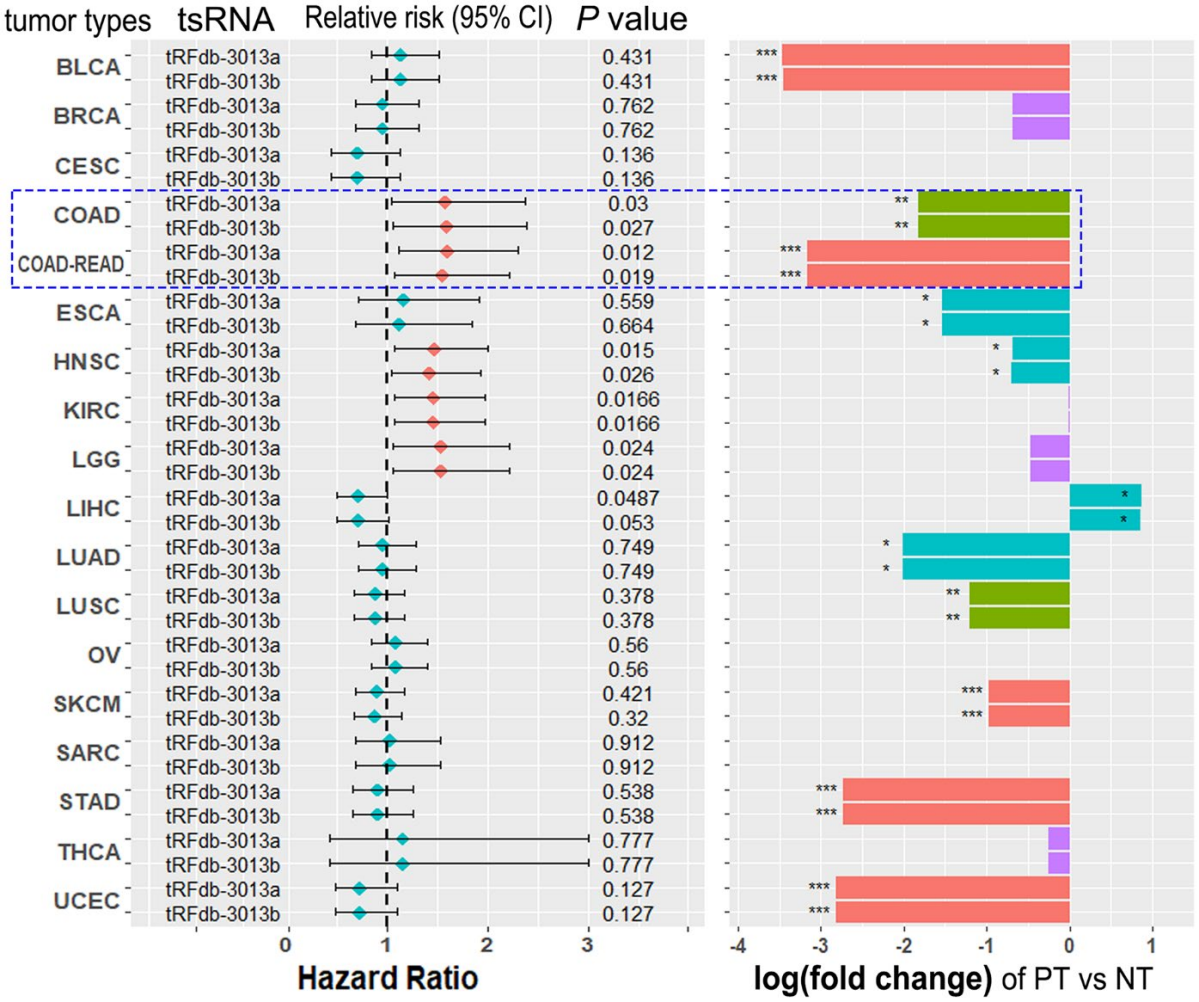
**The clinical significances of tRFdb-3013a and tRFdb-3013b within multiple different types of tumors.** (Left) The forest plots of Cox regression analysis with hazard ratio test based on tRFdb-3013a and tRFdb-3013b expressions within many types of tumors on TCGA dataset. (Right) The bar graph of differential expression analysis with a log2 fold-change test of tRFdb-3013a and tRFdb-3013b between primary tumor (TP) samples and non-tumor (NT) controls in multiple types of tumors.

Nevertheless, does the survival prognosis role of tRFdb-3013a/b specific to colorectal adenocarcinomas, and how about other types of tumors? In order to understand the clinical significances of tRFdb-3013a and tRFdb-3013b within multiple types of tumors, Cox regression analysis with hazard ratio test based on tRFdb-3013a/b expressions were performed within many types of tumors on TCGA datasets. As shown in the left of Figure 5, the forest plot described that tRFdb-3013a and tRFdb-3013b down-regulation were the risk factors associated with prognosis for five types of tumors in TCGA datasets, including COAD, READ, HNSC, KIRC, and LGG (all *P*≤0.03). Additionally, the differential expression analysis with log2 fold-change test of tRFdb-3013a and tRFdb-3013b were performed between primary tumor samples and paired non-tumor controls. As expected, the bar graphs showed that tRFdb-3013a/b expressions were markedly down-regulated in primary tumor samples compared to non-tumor controls for other six types of tumors (*P*<0.01, |log(FC)| >1, Figure 5-right). Taken together, the results implicated that only within colon and rectum adenocarcinoma, the expressions of tRFdb-3013a and tRFdb-3013b were both down-regulated, and could be served as the independent risk biomarkers for prognosis.

The correlations of tRFdb-3013a/b expression with primary clinical pathological characteristics were analyzed using *Chi*-square test, and visualized via hierarchical-clustering heatmaps [21]. As shown in Supplementary Figure 4, tRFdb-3013a/b might tend to decreased in the patients with lymphatic or vascular invasion present. However, no significant relationship was found between tRFdb-3013a/b expressions and other pathological characteristics, such as grade, gender, MLH1 silencing, methylation subtype, colon polyps and MSI status, etc. (all *P* > 0.05). Above all, findings demonstrated that tRFdb-3013a and tRFdb-3013b may serve as novel biomarkers for diagnosis and prognosis of colon adenocarcinomas.

### Biological insights of tRFdb-3013a/b on colon adenocarcinoma cells

**Figure 6:**
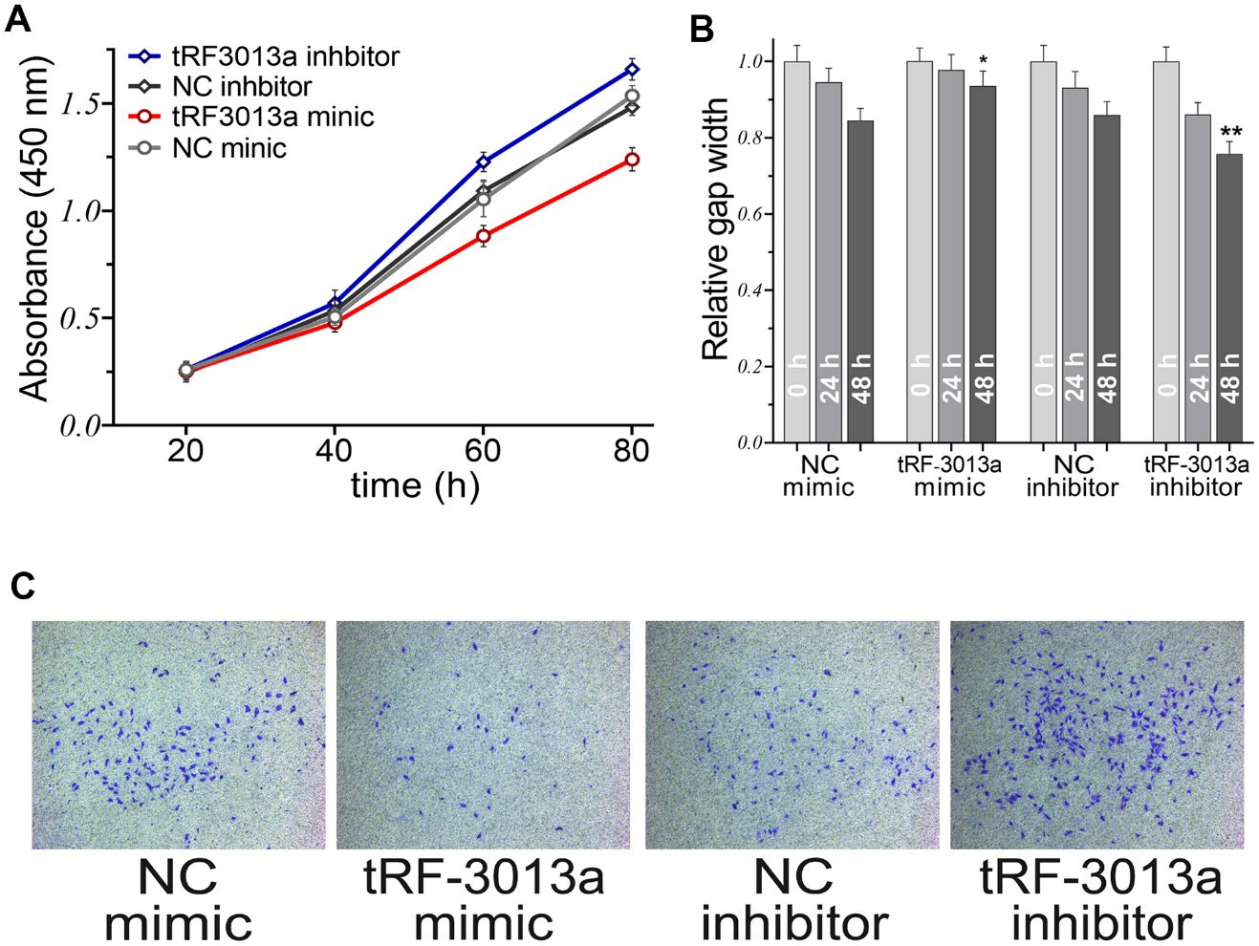
**The roles of tRFdb-3013a on cell proliferations, cell migration and invasion abilities of colon adenocarcinomas.** (**A**) CCK8 assay was performed to measure the cell proliferations of colon adenocarcinoma SW480 cell. (**B**) Relative gap analysis scratch assays were used to assess the migration ability of SW480 cell. (**C**) Transwell assays were performed to assess cell invasions of colon adenocarcinomas. ** *P* < 0.01, * *P* < 0.05. The “tRF-3013a” means as tRFdb-3013a, the “NC” means as negative control.

To further explore the roles of tRFdb-3013a/b on colon adenocarcinomas. The mimic or inhibitor was used to over-expressed or inhibit tRFdb-3013a in cancer cells (Supplementary Figure 5A). Cell proliferations of colon adenocarcinomas were detected using CCK8 assay, it showed that the proliferations were significantly suppressed in cells transfected tRFdb-3013a mimic compared to negative control (NC; both *P*<0.05; Figure 6A). Transwell and scratch assays were performed to test the effect of tRFdb-3013a on invasion and migration abilities of colon adenocarcinoma cells. The results displayed that migratory rates of cells transfected with tRFdb-3013a mimic were reduced in comparison with negative controls (Figure 6B and Supplementary Figure 5B); the numbers of invasive cells with tRFdb-3013a mimic were significantly reduced, and that cell numbers with tRFdb-3013a inhibitor were increased than those of negative controls (Figure 6C). These suggested that tRFdb-3013a might be effective to regulate the cell proliferations, cell migration and invasion abilities of colon adenocarcinomas.

**Figure 7:**
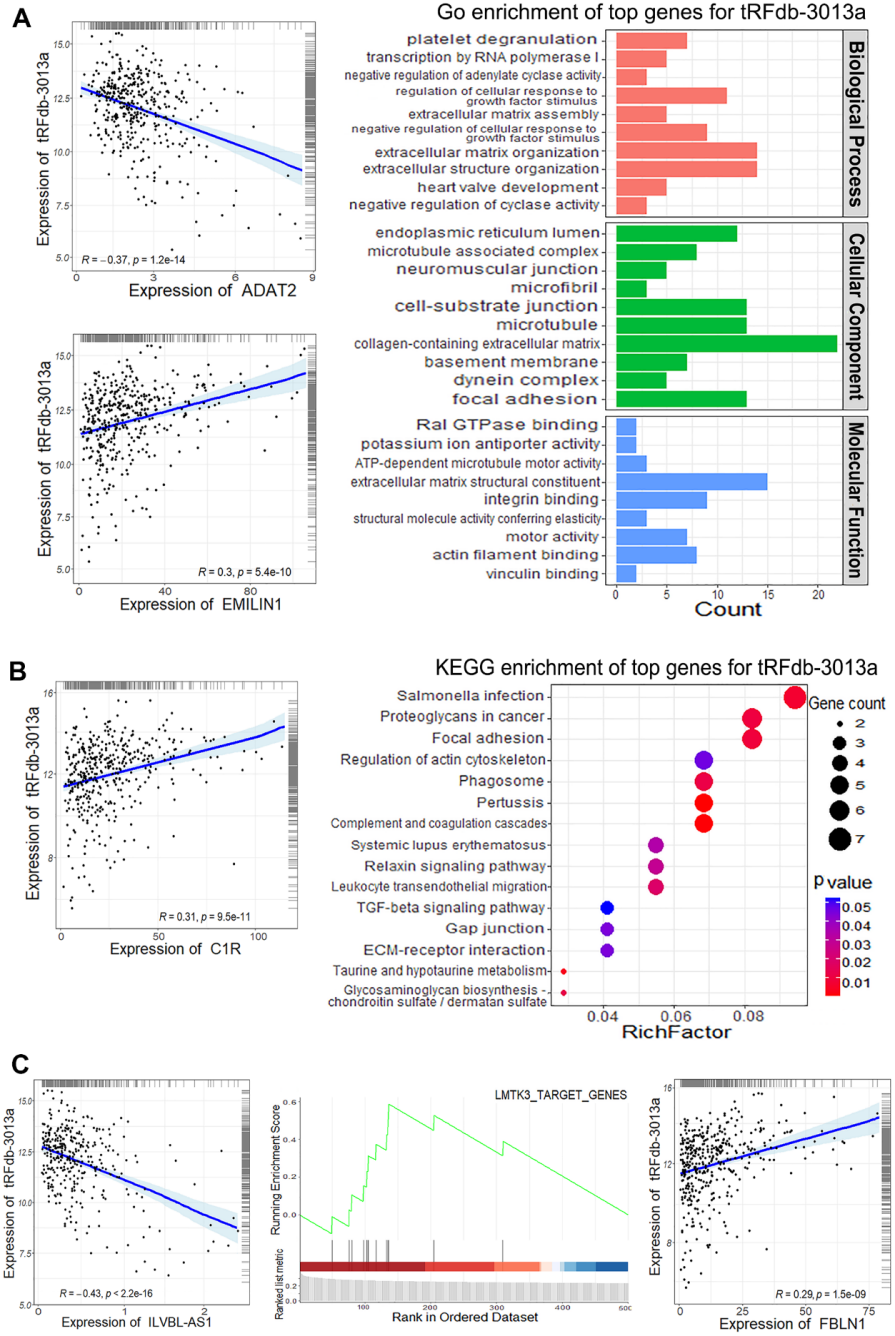
**The enrichment analysis of tRFdb-3013a related-genes within TCGA-COAD data.** (**A**) The top gene ontology (GO) terms, including biological process, cellular component and molecular function. (**B**) The top KEGG pathway for tRFdb-3013a related-genes. The correlation scatter-plots of tRFdb-3013a and its related-genes (ADAT2, EMILIN1, and C1R). (**C**) GSEA (gene set enrichment analysis) plots of the molecular signatures, the scatter plots for tRFdb-3013a and its related-genes (ILVBL-AS1, FBLN1).

Moreover, the enrichment analyses were performed to provide a biological insight concerning the roles of tRFdb-3013a/b, mRNA expression profiling of TCGA-COAD data was used to find the 500-top related-genes of tRFdb-3013a/b via *Spearman*’s correlation method. As shown in Figure 7A, for gene ontology (GO) analysis [22], some tRFdb-3013a related-genes were enriched in the specific GO terms, such as extracellular matrix organization, collagen-containing extracellular matrix, and extracellular matrix structural constituent (EMILIN1, ADAT2, FBLN1). The dot-plots (Figure 7B) displayed that tRFdb-3013a related-genes were enriched in several KEGG pathway, including phagosome (C1R). In the GSEA analyses [23] (Figure 7C), a molecular signature (LMTK3 target genes) was found to associated with tRFdb-3013a, such as FBLN1 and ILVBL-AS1 were both the tRFdb-3013a related-genes. The enrichment analysis of tRFdb-3013b were also conducted and presented in Supplementary Materials (Supplementary Figure 6). These data suggested that tRFdb-3013a/b might play a critical role in tumor progression of colon adenocarcinomas.

### The tRFdb-3013a/b target and regulate ST3GAL1 expression in colon adenocarcinomas

**Figure 8:**
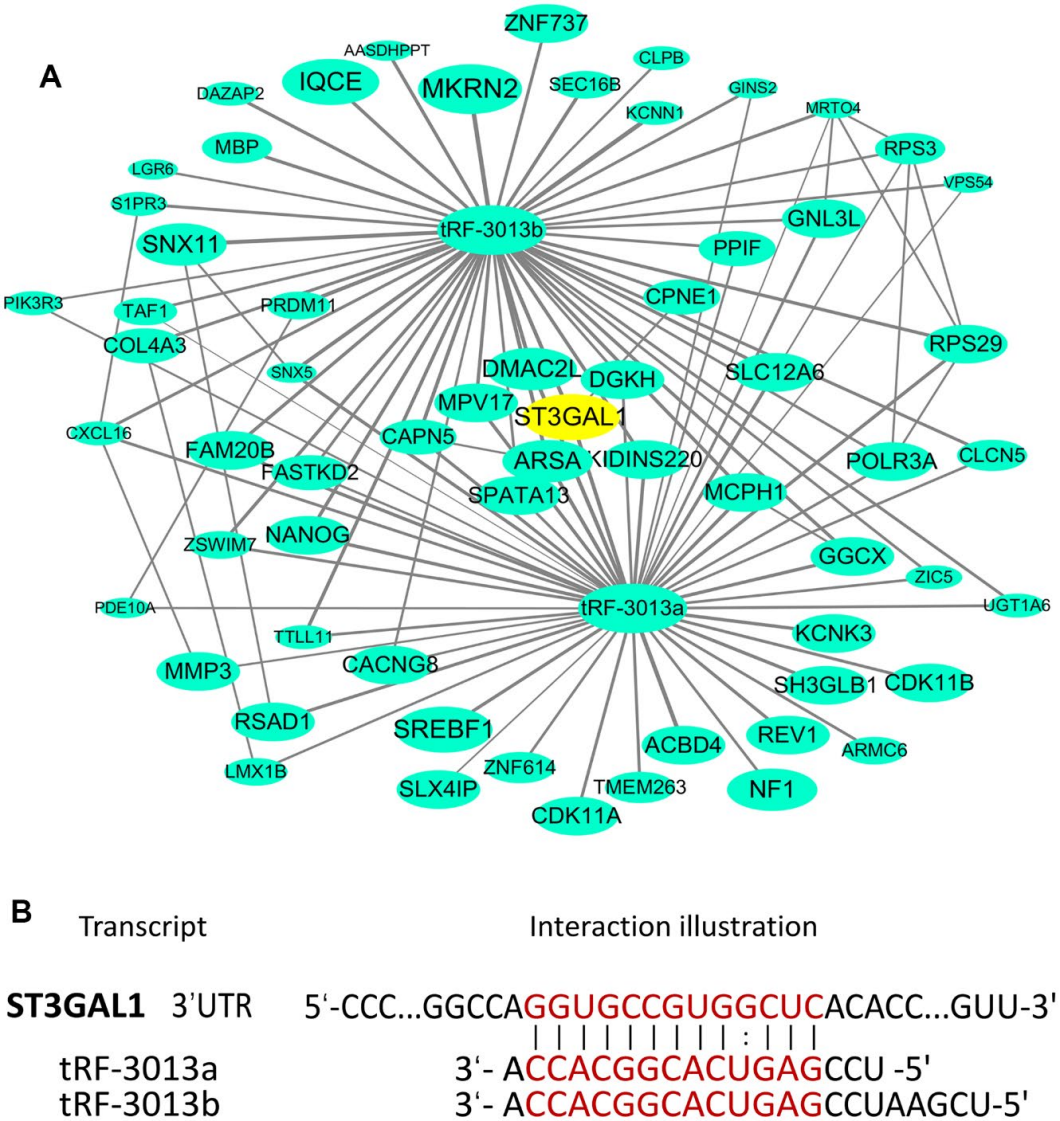
(**A**) The interactional network that involved tRFdb-3013a/b and their target genes based on tRFtarget database, the nodes represent target genes, and the lines represent the interaction relationships. (**B**) The binding sites of tRFdb-3013a/b in the 3’ UTR region of *ST3GAL1* based on bioinformatics prediction.

The researches have revealed that tRNA fragments could bind to argonaute (AGO) protein and lead to gene silencing by targeting the 3' untranslated region (3'UTR) of some mRNAs in a manner similar to miRNAs [5, 11]. Via using bioinformatics database and tools [24], many potential targets of tRFdb-3013a/b were predicted and selected out, the interaction relationship of tRFdb-3013a/b and their target genes were presented in Figure 8A.

**Figure 9:**
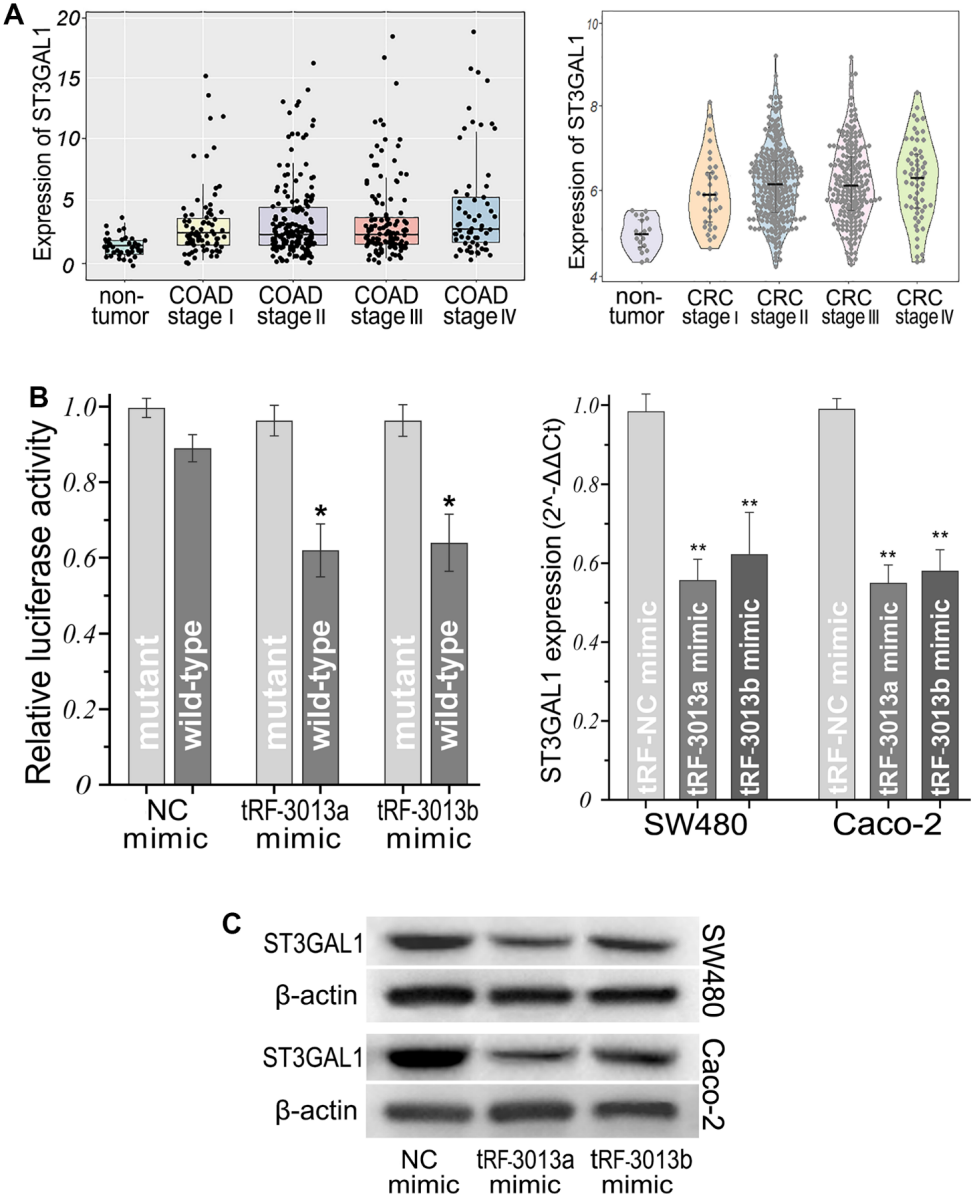
**ST3GAL1 was directly regulated by tRFdb-3013a and tRFdb-3013b in colon adenocarcinomas.** (**A**) ST3GAL1 was highly expressed in colon adenocarcinomas compared to non-tumor controls of TCGA-COAD and GSE39582 data (log_2_TPM, both *P* <0.01), COAD: colon adenocarcinomas, CRC: colorectal cancers. (**B**-left) The luciferase reporter assay of colon adenocarcinoma cell co-transfected with pmiR-RB-ST3GAL1 wild-type or mutant report vector, together with tRFdb-3013a/b or NC mimic. (**C**) Expression levels of ST3GAL1 mRNA (**B**-right) and protein in colon adenocarcinomas cells transfected with tRFdb-3013a/b mimic or control. ** *P* < 0.01, * *P* < 0.05. The “tRF-3013a/b” means as tRFdb-3013a/b, “NC” means as negative control.

Among these targets, we picked out a molecular with strong binding score, *ST3GAL1* gene, which encodes a type II membrane protein with sialyl-transferase activity [25]. The 3′UTR position of *ST3GAL1* mRNA contained a putative binding site of tRFdb-3013a/b (Figure 8B and Supplementary Figure 7). Dual-luciferase reporter assays were used to validate their direct targeting affinity, it showed that relative luciferase activity was decreased in the co-transfection of pmirRB-ST3GAL1 wild-type reporter and tRFdb-3013a/b mimic (both *P* < 0.05, Figure 9B-left), but no significant difference in the mutant reporter. Moreover, the analysis of TCGA-COAD and GSE39582 data showed that ST3GAL1 was highly expressed in colon adenocarcinomas compare to non-tumor controls (Figure 9A, both *P* <0.01), as mentioned above, tRFdb-3013a and tRFdb-3013b were conversely decreased in colon adenocarcinomas (Figure 2B). In addition, the experiments demonstrated that expression levels of ST3GAL1 mRNA and protein were down-regulated in colon adenocarcinoma cells transfected with tRFdb-3013a/b mimic (both *P* < 0.01, Figure 9B-right, 9C). These findings indicated tRFdb-3013a and tRFdb-3013b might directly target ST3GAL1 and regulate ST3GAL1 expressions in colon adenocarcinoma cells.

## DISCUSSION

Identifications and characterizations of tsRNAs are accelerated sharply in the last few years, due in large part to the emergence of next generation sequencing system-level approaches to molecular genetics [5]. In the present study, we firstly identified some tsRNAs within the sncRNA-seq datasets of colon adenocarcinoma samples, most of tsRNAs were derived from the 3′ end of mature tRNA or 3′ trailer regions of primary tRNA genes. Furthermore, a total of forty-two differentially expressed tsRNA genes were discriminated by bioinformatics analysis, of which fifteen were down-regulated and twenty-seven were up-regulated between colon adenocarcinomas and non-tumor controls. In our previous cytobiology study for microRNA, we have noted that it is easy to get the over-expression of microRNA with miRNA mimic, but hard to inhibit or decrease microRNA expression via miRNA inhibitor *in vitro*. Similarly, for the down-regulated tsRNA, we could make it over-expressed in cells via tsRNA mimic. Therefore, we selected several downregulated tsRNAs for further research. Among them, we picked out seven tsRNAs derived from tRNA-His-GUG, which was a histidyl-transfer-RNA [18]. There were two 3’-tRFs fragments (tRFdb-3013a, whose tDRnamer ID is tDR-60:76-His-GTG-1-M2; and tRFdb-3013b, whose tDRnamer ID is tDR-55:76-His-GTG-1-M2 [26]) can re-map to the cleavage of 3′ end of mature tRNA-His-GUG, the expressions of tRFdb-3013a and tRFdb-3013b were markedly down-regulated in colorectal adenocarcinomas compared to non-tumor controls. KM curve analysis displayed that clinical survival of colon adenocarcinoma patients with low tRFdb-3013a/b expressions were significantly worse than that of the high-expression patients, it suggested that down-regulated tRFdb-3013a and tRFdb-3013b were associated with poor survival prognosis of patients with colon adenocarcinoma and/or rectum adenocarcinoma. In the further analyses of other tumor types from TCGA datasets, however, only within colon adenocarcinoma (COAD) and/or rectum adenocarcinoma (READ), the expressions of tRFdb-3013a and tRFdb-3013b were both down-regulated, and could be served as risk biomarkers for prognosis, not for other type tumors. Further analyses revealed that tRFdb-3013a/b expression might tend to decrease in the patients with lymphatic or vascular invasion present. These findings implied that tRFdb-3013a and tRFdb-3013b might serve as novel biomarkers for diagnosis and prognosis of colorectal adenocarcinomas.

Growing evidences have demonstrated that dysregulated tRNA fragments may contribute to various biological processes of cancer development and progression [13]. For example, Tao et al. have revealed the expression profiles of tsRNAs in human colorectal cancer (CRC) specimens via sequencing analyses, and identified a specific tsRNA derived from the internal of mature histidyl-tRNA, 5'tiRNA-His-GTG (its base sequence is GCCGTGATCGTATAGTGGTTAGTACTCTGCGTTGT), which is up-regulated in CRC tissues [11]. Then, some experiments have revealed that an inhibition of 5'tiRNA-His-GTG suppressed cell proliferations and colony formations, induced cell apoptosis, and affected tumor growth in nude mice, these indicate that 5'tiRNA-His-GTG may act an oncogenic role in the progression of colorectal cancers [11]. In glioma, Ren et al. have determined the differential expressed tsRNAs between tumor tissues with matched non-tumor controls, and found some tRNA-Cys-GCA derived tsRNAs were significantly decreased in tissues from gliomas. Moreover, lower expression of tRNA-Cys-GCA derived tsRNA is associated with poor survival outcome of glioma patients [10]. In present study, certain cyto-biology assays were performed to provide a biological insight concerning the roles of tRFdb-3013a on colon adenocarcinoma cells. It demonstrated that tRFdb-3013a over-expression suppressed cell proliferations, migration and invasion abilities of colon adenocarcinomas *in vitro*. Moreover, the enrichment analysis showed tRFdb-3013a was associated with specific GO terms, such as extracellular matrix organization, collagen-containing extracellular matrix, and extracellular matrix structural constituent (EMILIN1, ADAT2, FBLN1); the tRFdb-3013a related-genes might refer to phagosome pathway (C1R), as well as the GSEA molecular signature genes (FBLN1 and ILVBL-AS1). These results indicated that down-regulated tRFdb-3013a might act a suppressive role in cell migration and invasion of colon adenocarcinomas via specific signaling pathway.

Though the biological function of tsRNAs is complex and require further elucidation, current knowledge of their function has been a key focus on cancer researches. Previous researches have revealed that tsRNAs could bind to AGO proteins and play a critical role by directly sponging the 3'UTRs of some mRNAs in a manner similar to miRNAs [5]. For example, Goodarzi et al. have identified the new class of tsRNAs derived from several tRNA through hypoxic stress induction, these tsRNAs could bind to the 3'UTRs of *YBX1* gene, which encoded an RNA-binding protein, then suppressed the stability of multiple oncogenic transcripts, including *EIF4EBP1* and *AKT1*, results in the tumor-suppressive and metastasis-suppressive activities in breast cancers [12]. Our bioinformatics analyses displayed the interaction relationships of tRFdb-3013a/b and their predicted target genes, and found that tRFdb-3013a/b could bind to the 3’UTR of *ST3GAL1*, which was highly expressed in colon adenocarcinomas. ST3GAL1 is a membrane protein with sialyl-transferase activity, which has been reported to promote tumor metastasis and invasion by previous study [25]. The luciferase reporter assay showed that tRFdb-3013a/b could directly target 3′UTR of ST3GAL1 and decrease its relative luciferase activity. In addition, tRFdb-3013a over-expressions could down-regulate the expression levels of ST3GAL1 protein and mRNA in colon adenocarcinoma cells. These results suggest tRFdb-3013a and tRFdb-3013b might directly target the 3′UTR of ST3GAL1 and regulate ST3GAL1 expressions in colon adenocarcinomas.

## CONCLUSIONS

Taken together, our investigation identified the tsRNAs profiles, particularly several tsRNAs derived from tRNA-His-GUG, in the sncRNA-seq data of colon adenocarcinoma samples. Among these, tRFdb-3013a and tRFdb-3013b were significantly down-regulated in colon and rectum adenocarcinomas, the expressions of tRFdb-3013a/b were associated with clinical survival prognosis of colon and rectum adenocarcinoma patients. Over-expression of tRFdb-3013a inhibited cell proliferations, cell migrations and invasions of colon adenocarcinomas. The down-regulated tRFdb-3013a might act an anti-oncogene role in colon adenocarcinomas via specific signaling pathway. Additionally, tRFdb-3013a/b could directly target the 3′UTR of ST3GAL1 and regulate ST3GAL1 expressions in colon adenocarcinomas. These results implied that tRFdb-3013a/b may play an important role in the progression of colon adenocarcinomas, and could be explored as novel biomarkers for diagnosis and prognosis of colon and rectum adenocarcinomas.

## MATERIALS AND METHODS

### Bioinformatic analysis

The RNA expression profiles of TCGA datasets were downloaded and processed using TCGAbiolinks package [8]. The differential expression analysis between paired colon adenocarcinoma samples and non-tumor controls was performed with the limma package [33]. An absolute log2 fold-change (|log_2_FC|) more than one and *P*-value less than 0.05 were set as cut-off point. Hierarchical clustering of differentially expressed tsRNA genes was performed based on expression values to verify the difference between paired colon adenocarcinomas and non-tumors [19]. Visualization of the tsRNAs including volcano plots and heatmaps was performed with the ggplot2 [34] and pheatmap packages of R, respectively. Survival analysis with Kaplan-Meier (KM) curves plus the log-rank test was performed via the R survival and survminer packages. Hierarchical cluster analysis was performed on tsRNAs expression and primary pathology characteristics, its corresponding heatmap was visualized by pheatmap package (https://cran.r-project.org/web/packages/pheatmap/). *Spearman’s* correlation was calculated for each pair of tsRNA and mRNA, its scatter diagrams were plotted by ggplot2 and ggpubr package in R software [34]. Some enrichment analyses were performed to provide biology views that regard tRFdb-3013a/b-related genes, the molecular signatures database (MSigDB) was used as gene set for gene set enrichment analysis (GSEA) [23]. Gene ontology, KEGG pathway, and GSEA analysis were visualized by clusterProfiler and enrichplot packages [22, 35]. The computational binding interactions between tsRNA and targets were predicted by tRFtarget databases [24]. The interaction networks of top-50 targets and tRFdb-3013a/b were performed and visualized via Cytoscape (v3.7.2).

### Cell culture and transfections

Human colon adenocarcinoma cell lines SW480 and Caco-2 were purchased from the Cell Bank of Chinese Academy of Sciences (Shanghai, China). The cells were cultured in Leibovitz's L-15 medium or Eagle's Minimum Essential Medium (MEM; Servicebio, Wuhan, China) supplemented with 10% or 20% fetal bovine serum (FBS; Servicebio) at 37° C in a humidified incubator with 5 % CO_2_. The cell lines at 50–70% confluences were transfected with micromolar either mimic for tRFdb-3013a/b (tRF-3013a/b mimic), or inhibitor for tRFdb-3013a (tRF-3013a inhibitor), or negative controls (NC mimic, NC inhibitor) using the Lipofectamine RNAimax reagent (Invitrogen, Thermo Fisher Scientific, USA). The mimic and inhibitor for tRFdb-3013a/b and negative controls were purchased from RiboBio BioTech (Guangzhou, China).

### RNA extraction and qRT-PCR analysis

Total RNAs were extracted from cultured cells using the RNAex reagent (AGbio, Changsha, China). One microgram total RNAs of each sample were reversely transcribed into cDNA under standard conditions by using PrimeScript RT reagent Kit with gDNA Eraser (Takara, Shiga, Japan). Quantitative real-time polymerase chain reaction (qRT-PCR) was performed with the SYBR^®^ Premix DimerEraser™ (Takara) on the CFX connect real-time system (Bio-Rad, USA). The Bulge-Loop miRNA qRT-PCR Stater Kit (RiboBio, China) with specific stem-loop RT primers was used to quantify the expression of tsRNAs, following the manufacturer's protocol. RNU6 (U6) was used as the internal control for tsRNAs template normalization, and ACTB (β-actin) for mRNA template normalization. Relative quantification of gene expression was calculated by the comparative cycle-threshold (CT) method [19]. Stem-loop primers for tRFdb-3013a/b, and RNU6 were designed and synthesized by RiboBio BioTech (Guangzhou, China). The general primers for ST3GAL1 and ACTB were designed and synthesized by Sangon Biotech (Shanghai, China), and the primers sequences were listed in Supplementary Table 2.

### Protein extraction and Western blot

Total proteins were extracted from cultured cells using RIPA buffer (Beyotime, Shanghai, China) and the protein concentrations were determined using a BCA protein assay kit (Servicebio). The specific primary antibody (anti-ST3GAL1 antibody diluted at 1:1000, Abcam, USA; anti-β-actin antibody diluted at 1:1500, Sigma-Aldrich, USA). The β-actin served as an endogenous protein for normalization. The protein bands were visualized using ChemiScope S6 imaging system and processing software (Clinx, Shanghai, China).

### Cell proliferation, migration and invasion assays

Cell proliferation assays were performed with Cell Counting Kit-8 (CCK-8; Selleck, USA), the absorbance (A) in each well was measured at 450 nm with multimode Microplate Reader (Varioskan LUX, Thermo Fisher Scientific). Cell migration and invasion abilities were tested via the scratch and transwell assays, the images of scratch gap and invasion cells were recorded and photographed using Nikon eclipse Ti-S microscope (Tokyo, Japan).

### Luciferase reporter assay

The wild-type or mutant sequences of ST3GAL1 3’UTR containing putative tRFdb-3013a and tRFdb-3013b binding sites were subcloned into pmir-RB-Report vector (RiboBio, China). SW480 cells were co-transfected with pmir-RB-Report vector plus or without tRFdb-3013a/b. The luciferase activity was measured using the Dual-luciferase Reporter Assay System (Promega, USA).

### Statistical analysis

The statistical analyses were performed with the R software (https://www.r-project.org/), GraphPad Prism version 8.0 (GraphPad Software, Inc., USA) was used for graphing and analysis. Data were exhibited as means ± standard deviation (SD). The *Chi*-square test was used to compare the categorical variables. Regarding the numerical variables, statistical significance of differences between two groups was assessed using two-sided Student’s t test; and comparisons of multiple groups were made by one-way analysis of variance. All experiments were performed in triplicate and *P* values less than 0.05 were considered a statistically significant difference.

### Data availability statement

The present study used secondary data which are available in the public domain. The dataset has no identifiable information of the survey participants. All data and materials used during the current study are available from the corresponding author on reasonable request. Some limitations about tsRNA identifications were elaborated and described in Supplementary Materials. 

## Supplementary Material

Supplementary Methods

Supplementary Figures

Supplementary Table 1

Supplementary Table 2

Supplementary Table 3

Supplementary Table 4

Supplementary Table 5
