# Supplementary material for: The tsRNAs (tRFdb-3013a/b) serve as novel biomarkers for colon adenocarcinomas
Source: Aging (Albany NY). 2024 Mar 6;16(5):4299–326. doi: 10.18632/aging.205590 (PMC10968714; doi:10.18632/aging.205590)
Supplement: Supplementary Methods [file aging-16-205590-s001.pdf]

SUPPLEMENTARY METHODS

Data collection and processing

The sequences of human tsRNAs were taken from tRFdb database (http://genome.bioch.virginia.edu/trfdb/) and tRFexplorer database (https://trfexplorer.cloud/), including different types of tsRNA fragments: 5′-tRFs, 3′-tRFs, 5′U-tRFs and tRF-1 [1, 2]. The hg19 reference genome annotations and corresponding sequences of tRNAs in humans were taken from GtRNAdb (genomic tRNA database, http://gtrnadb.ucsc.edu/) [3]. Then, a custom annotation of the reference human gene containing only known tsRNAs was assembled. The small non-coding RNA sequencing (sncRNA-seq) data of colon adenocarcinoma (COAD) samples and rectum adenocarcinoma (READ) samples on TCGA, the RNA sequencing datasets of TCGA-COAD and TCGA-READ samples, and the corresponding patient clinical information were retrieved from GDC data portal [4]. The primary clinical and molecular pathology characteristics of COAD patients were listed in Supplementary Table 1. The gene expression profiles of GSE39582 were downloaded from the Gene Expression Omnibus (http://www.ncbi.nlm.nih.gov/geo/) database, the gene expression microarrays are based on Affymetrix Human Genome U133 Plus 2.0 Array platform (Affymetrix, USA) [5].

A conservative pipeline was implemented to get an accurate estimation of tsRNAs expression as demonstrated previously [2], the data processing and flow chart for tsRNAs identification are showed in Supplementary Figure 1A. The quality controls and data filtering of raw files on sncRNA-seq data were pre-processed via using Cutadapt and FastQC method. Then, clean reads of small RNA sequencing were re-mapped to the reference human genome (GRCh37 /hg19) and the sequences of tsRNAs annotation file via applying Bowtie software [6]. After alignment, only the mapped reads could be quantified to count the number of reads belonging to each of the candidate tsRNAs with HTSeq software [7]. Finally, the expression value of tsRNAs was calculated and normalized as transcripts per million reads (TPM) of total raw counts [8], and the average expression values less than one log2TPM were filtered to eliminate random degradation sequences.

The clinical specimens

Fifteen carcinoma tissues and five para-carcinoma tissues were obtained from patients diagnosed with colon adenocarcinomas undergoing surgical resections at the Department of Gastrointestinal Surgery, the First Affiliated Hospital of Chongqing Medical University from February 2022 to October 2023. After excisions, tissues were immediately frozen in liquid nitrogen for subsequent use. This study was approved by the Ethics Committees of Chongqing Medical University and the patients provided written informed consent.

Northern blotting

Total RNA was isolated from the cells using TRIzol reagent (Invitrogen, USA). Before loading, the RNA samples were denatured at 65° C for 5 min and chilled on ice immediately. The samples were separated on a 15% denaturing polyacrylamide gel and electrophoretically transferred to a charged nylon membrane (Labselect, China). Following cross-linking and pre-hybridization, the RNA was hybridized with the corresponding 3′-digoxigenin (DIG)-labeled DNA probe at 42° C overnight [17]. After washing and blocking, the membrane was incubated with anti-DIG antibody solution (Servicebio, China). The chemiluminescence signal was captured and analyzed via a ChemiScopeS6 imaging system (Clinx, China). The DIG-labeled tRFdb-3013a/b probe (5’-TGGTGCCGTGACTCGGA-3′), the DIG-labeled tRNA-His-GUG probe (5’-CGGCCACAACGCAGAGTACT-3′) and the 5S rRNA probe (as an internal control) were synthesized by Sangon Biotech (Shanghai, China).

Limitations

In this study, tsRNAs refer to that fragments derived from the tRNAs based on the GtRNAdb, tRFdb and tRFexplorer databases by a conservative identification pipeline. With computational approaches, we determined the tsRNAs expression profile within the TCGA-COAD dataset. Particularly, our bioinformatic analysis has focused on the role of tRFdb-3013a and tRFdb-3013b, which are two of the tsRNA fragments derived from tRNA-His-GTG gene. Furthermore, there should be many ways, through diversification of methods, addressing the tRNA-derived fragments mining with a conservative pipeline. Recently, a tRNA-derived fragments (tRFs) repository has been released, MINTbase v2.0, in which more than ten thousand tRFs were mined from the TCGA datasets with their own deterministic and exhaustive pipeline.

There were some differences or limitations between some naming system of tsRNA, while compared to the profiles of tsRNAs that identified through tDRnamer and MINTbase method and our identification pipeline within COAD dataset. Take the example of tRFdb-3013a and tRFdb-3013b, two of the fragments derived from tRNA-His-GTG gene; as shown in the Supplementary Figure 8A, 8B, these are a total of

forty-eight fragments derived from the 3′ end of mature tRNA-His-GTG in tDRnamer, among them, twenty fragment isoforms could be precisely aligned to the sequence of tRFdb-3013a, and twenty-eight fragment isoforms can be aligned to the sequence of tRFdb-3013b. Actually, two tRF isoforms (tDR-60:76-His-GTG-1-M2, whose MINTbase ID is tRF-17-8US5652; and tDR-55:76-His-GTG-1-M2, whose MINTbase ID is tRF-22-WB8US5652) were two of the most abundant fragments that identified from the TCGA datasets, and may be seen as a typical representative of tRFdb-3013a and tRFdb-3013b. Moreover, the primers for tRFdb-3013a/b detection with stem-loop qRT-PCR assay, were designed to match the sequence (TCCGAGTCACGGCA or TCGAATCCGAGTCACGGCA), hence the expression levels should refer to the fold-change of tRFdb-3013a/b, which may be detected, not only one fragment isoform, in the qRT-PCR experiments. In this case, tsRNAs generally refer to the small RNAs derived from the tRNAs, instead of the tRFs or tRNA isoforms with highly similar sequences, to avoid overestimation and artifacts that are presented in tDRnamer and MINTbase.

As mentioned in the GtRNAdb, there are about 64 classes of tRNAs which correspond to twenty-two kinds of amino acids in the human genome, since each amino acid has many different anticodons such as histidine, which has two specific anticodons and that makes up two isotypes of tRNAs (tRNA-His-ATG and tRNA-His-GTG). Hence it is conservatively estimated that not more than four tsRNAs derived from the 3′ end of two mature tRNA isotypes for histidine. Undoubtedly, it will be more complex in the human genome transcriptional system, of the tRNA-His-GTG isotypes, twenty tRNA isoforms (including tRNA-His-GTG-1-1, tRNA-His-GTG-1-2, tRNA-His-GTG-1-6, tRNA-His-GTG-1-7, and tRNA-His-GTG-1-8, etc.) were identified with almost similar sequences, and sometimes only individual bases differ between these twenty sequences (As shown in Figure 2A). Even though with respect to tDRnamer and MINTbase which contain thousands of tRFs in TCGA datasets, it is not surprising that only two hundred tsRNAs were identified in COAD samples based on the GtRNAdb, tRFdb and tRFexplorer databases with our conservative pipeline.

Supplementary References

1. Kumar P, Mudunuri SB, Anaya J, Dutta A. tRFdb: a database for transfer RNA fragments. Nucleic Acids Res. 2015; 43:D141–5. https://doi.org/10.1093/nar/gku1138 PMID:25392422

2. La Ferlita A, Alaimo S, Veneziano D, Nigita G, Balatti V, Croce CM, Ferro A, Pulvirenti A. Identification of tRNA-derived ncRNAs in TCGA and NCI-60 panel cell lines and development of the public database tRFexplorer. Database (Oxford). 2019; 2019:baz115. https://doi.org/10.1093/database/baz115 PMID:31735953

3. Chan PP, Lowe TM. GtRNAdb 2.0: an expanded database of transfer RNA genes identified in complete and draft genomes. Nucleic Acids Res. 2016; 44:D184–9. https://doi.org/10.1093/nar/gkv1309 PMID:26673694

4. Hutter C, Zenklusen JC. The Cancer Genome Atlas: Creating Lasting Value beyond Its Data. Cell. 2018; 173:283–5. https://doi.org/10.1016/j.cell.2018.03.042 PMID:29625045

5. Barrett T, Wilhite SE, Ledoux P, Evangelista C, Kim IF, Tomashevsky M, Marshall KA, Phillippy KH, Sherman PM, Holko M, Yefanov A, Lee H, Zhang N, et al. NCBI GEO: archive for functional genomics data sets--update. Nucleic Acids Res. 2013; 41:D991–5. https://doi.org/10.1093/nar/gks1193 PMID:23193258

6. Langmead B, Trapnell C, Pop M, Salzberg SL. Ultrafast and memory-efficient alignment of short DNA sequences to the human genome. Genome Biol. 2009; 10:R25. https://doi.org/10.1186/gb-2009-10-3-r25 PMID:19261174

7. Anders S, Pyl PT, Huber W. HTSeq--a Python framework to work with high-throughput sequencing data. Bioinformatics. 2015; 31:166–9. https://doi.org/10.1093/bioinformatics/btu638 PMID:25260700

8. Liao Y, Smyth GK, Shi W. featureCounts: an efficient general purpose program for assigning sequence reads to genomic features. Bioinformatics. 2014; 30:923–30. https://doi.org/10.1093/bioinformatics/btt656 PMID:24227677

9. Colaprico A, Silva TC, Olsen C, Garofano L, Cava C, Garolini D, Sabedot TS, Malta TM, Pagnotta SM, Castiglioni I, Ceccarelli M, Bontempi G, Noushmehr H. TCGAbiolinks: an R/Bioconductor package for integrative analysis of TCGA data. Nucleic Acids Res. 2016; 44:e71. https://doi.org/10.1093/nar/gkv1507 PMID:26704973

10. Ritchie ME, Phipson B, Wu D, Hu Y, Law CW, Shi W, Smyth GK. limma powers differential expression analyses for RNA-sequencing and microarray studies. Nucleic Acids Res. 2015; 43:e47. https://doi.org/10.1093/nar/gkv007 PMID:25605792

11. Xu B, Liang J, Zou H, Wang J, Xiong Y, Pei J.

Identification of Novel tRNA-Leu-CAA-Derived tsRNAs for the Diagnosis and Prognosis of Diffuse Gliomas. Cancer Manag Res. 2022; 14:2609–23.
https://doi.org/10.2147/CMAR.S367020
PMID:36072386

12. Wickham H. ggplot2: Elegant Graphics for Data Analysis. Use R!,. (Cham: Springer International Publishing: Imprint: Springer). 2016:1.
https://doi.org/10.1007/978-3-319-24277-4

13. Liberzon A, Birger C, Thorvaldsdóttir H, Ghandi M, Mesirov JP, Tamayo P. The Molecular Signatures Database (MSigDB) hallmark gene set collection. Cell Syst. 2015; 1:417–25.
https://doi.org/10.1016/j.cels.2015.12.004
PMID:26771021

14. Kanehisa M, Furumichi M, Tanabe M, Sato Y, Morishima K. KEGG: new perspectives on genomes, pathways, diseases and drugs. Nucleic Acids Res. 2017; 45:D353–61.
https://doi.org/10.1093/nar/gkw1092
PMID:27899662

15. Wu T, Hu E, Xu S, Chen M, Guo P, Dai Z, Feng T, Zhou L, Tang W, Zhan L, Fu X, Liu S, Bo X, Yu G. clusterProfiler 4.0: A universal enrichment tool for interpreting omics data. Innovation (Camb). 2021; 2:100141.
https://doi.org/10.1016/j.xinn.2021.100141
PMID:34557778

16. Li N, Shan N, Lu L, Wang Z. tRFtarget: a database for transfer RNA-derived fragment targets. Nucleic Acids Res. 2021; 49:D254–60.
https://doi.org/10.1093/nar/gkaa831
PMID:33035346

17. Martinho C, Lopez-Gomollon S. Detection of MicroRNAs by Northern Blot. Methods Mol Biol. 2023; 2630:47–66.
https://doi.org/10.1007/978-1-0716-2982-6_4
PMID:36689175
