# Supplementary material for: The tsRNAs (tRFdb-3013a/b) serve as novel biomarkers for colon adenocarcinomas
Source: Aging (Albany NY). 2024 Mar 6;16(5):4299–326. doi: 10.18632/aging.205590 (PMC10968714; doi:10.18632/aging.205590)
Supplement: Supplementary Figures [file aging-16-205590-s002.pdf]

SUPPLEMENTARY FIGURES

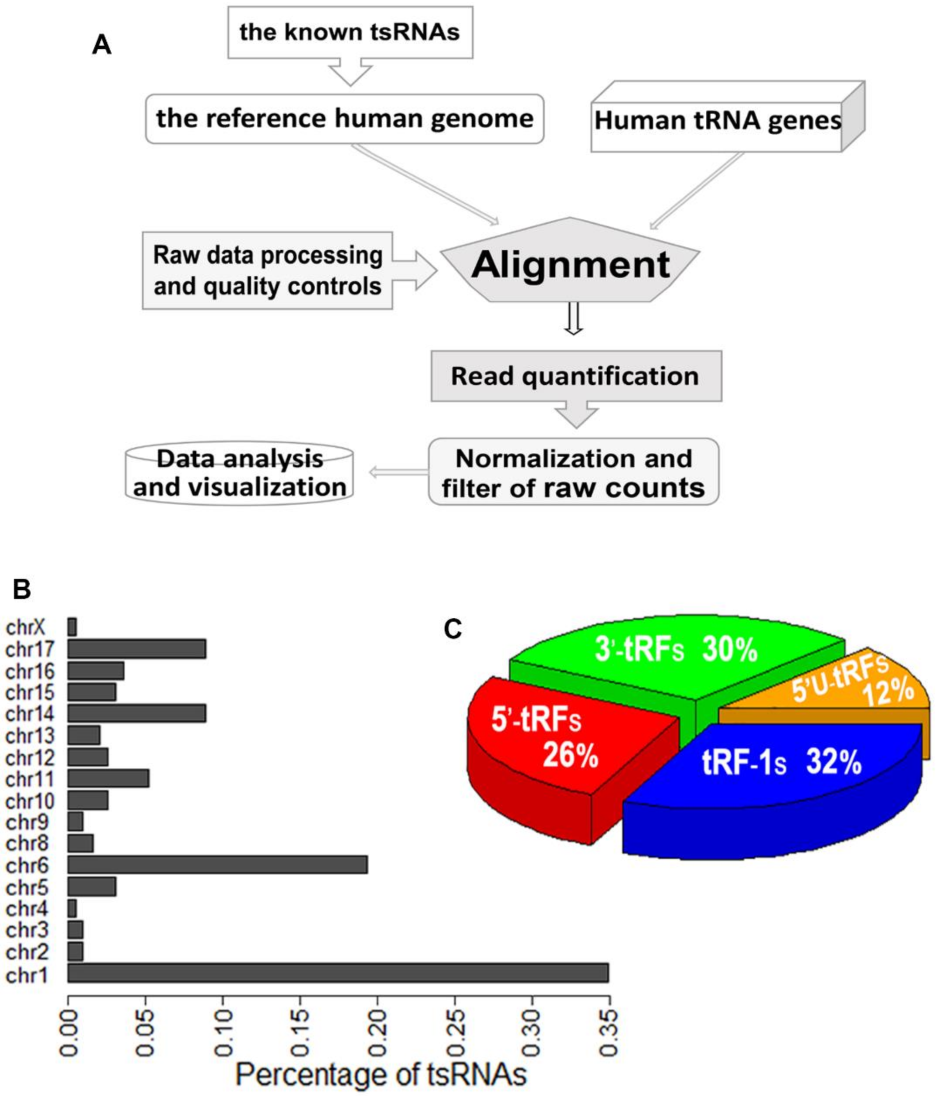

Supplementary Figure 1. A summary of tsRNAs identification and characterization. (**A**) The flow chart of data processing and tsRNAs identification pipeline. (**C**) The characterization of tsRNA corresponding derived tRNA gene sources and (**B**) the chromosome locations statistical analysis.

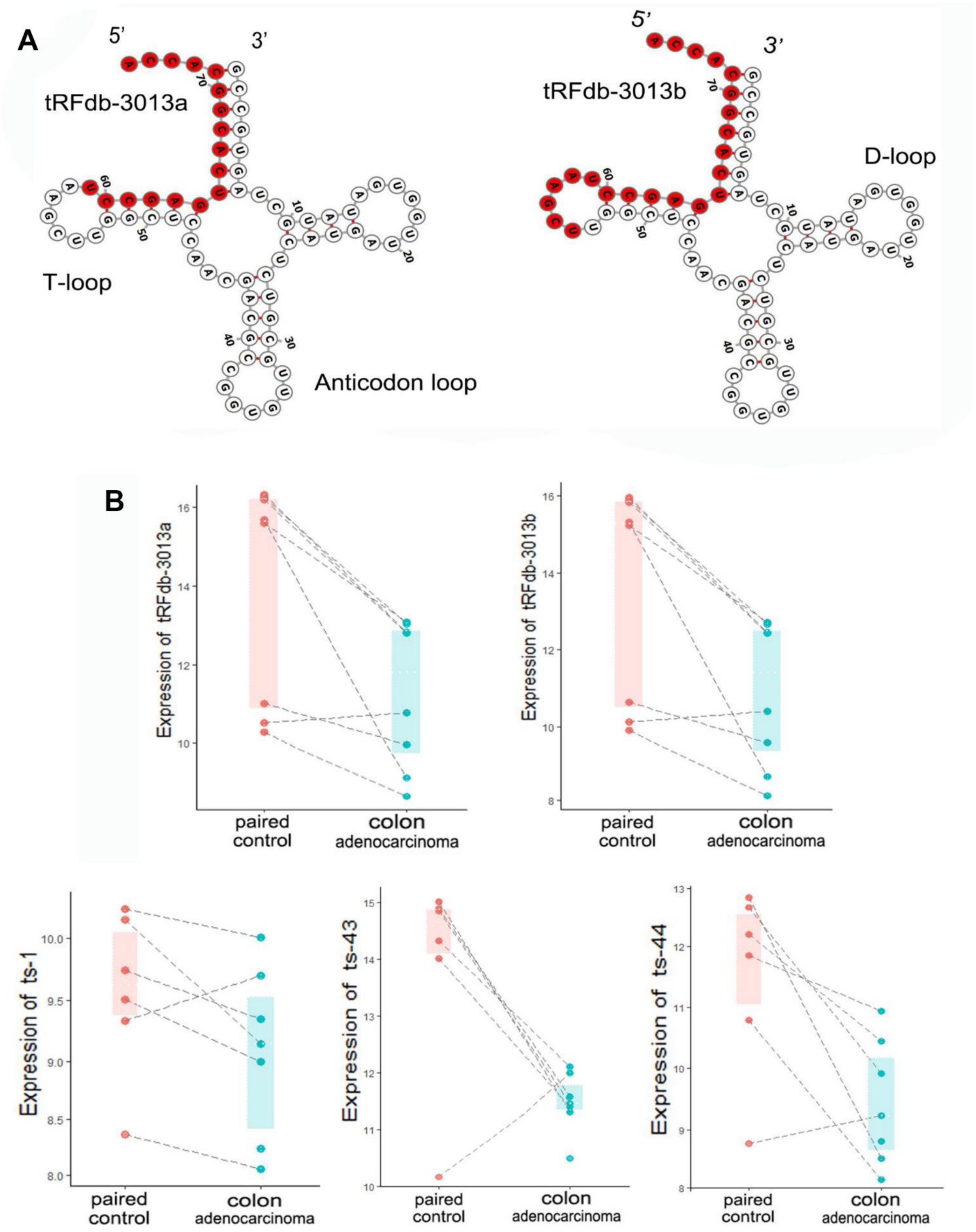

Supplementary Figure 2. (**A**) Representative secondary structure of tRNA-His-GUG and its derived fragments (tRFdb-3013a and tRFdb-3013b). (**B**) The expression patterns of several tRNA-His-GTG derived tsRNA fragments (including tRFdb-3013a, tRFdb-3013b, ts-1, ts-43 and ts-44) in paired colon adenocarcinomas and non-tumor controls of TCGA-COAD datasets.

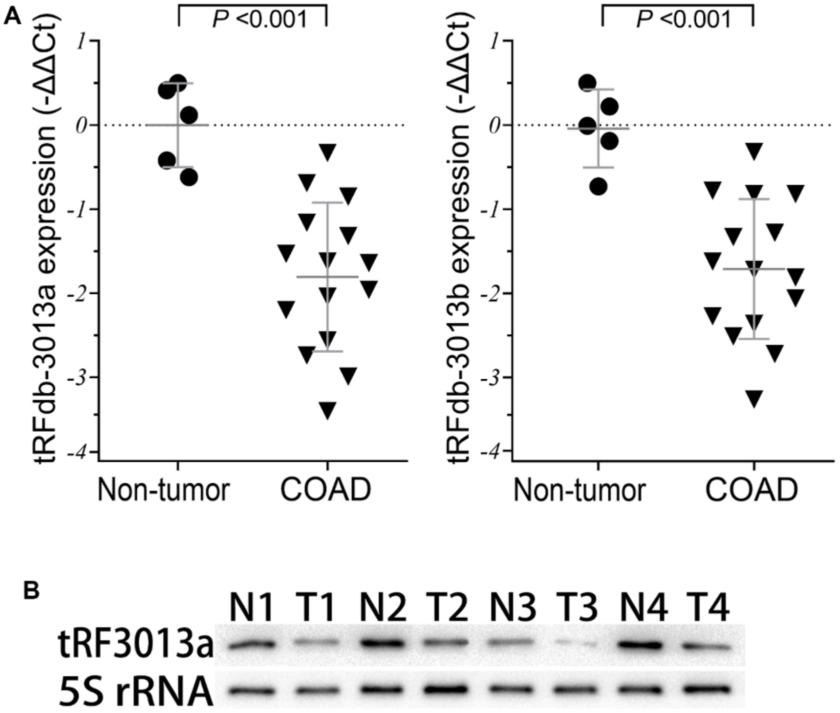

Supplementary Figure 3. (**A**) The boxplot of tRFdb-3013a/b expression between non-tumor controls and colon adenocarcinomas with the stem-loop tsRNA qRT-PCR analysis. (**B**) The Northern blotting analysis of tRFdb-3013a (tRF3013a) between non-tumor controls (N1-4) and colon adenocarcinoma (T1-4) samples (all P <0.01).

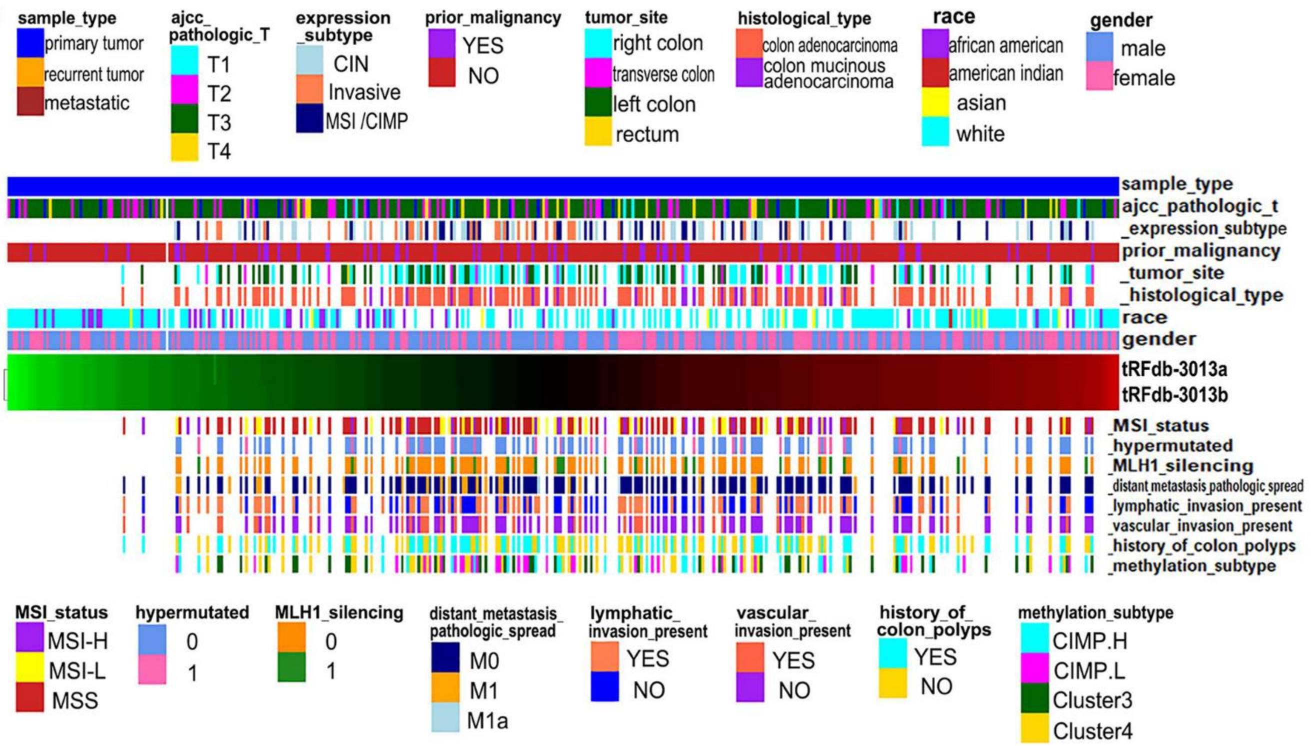

Supplementary Figure 4. The correlation analysis of tRFdb-3013a/b expression with the primary pathology characteristics. Hierarchical clustering heatmap of tRFdb-3013a/b expression and the pathology characteristics parameters.

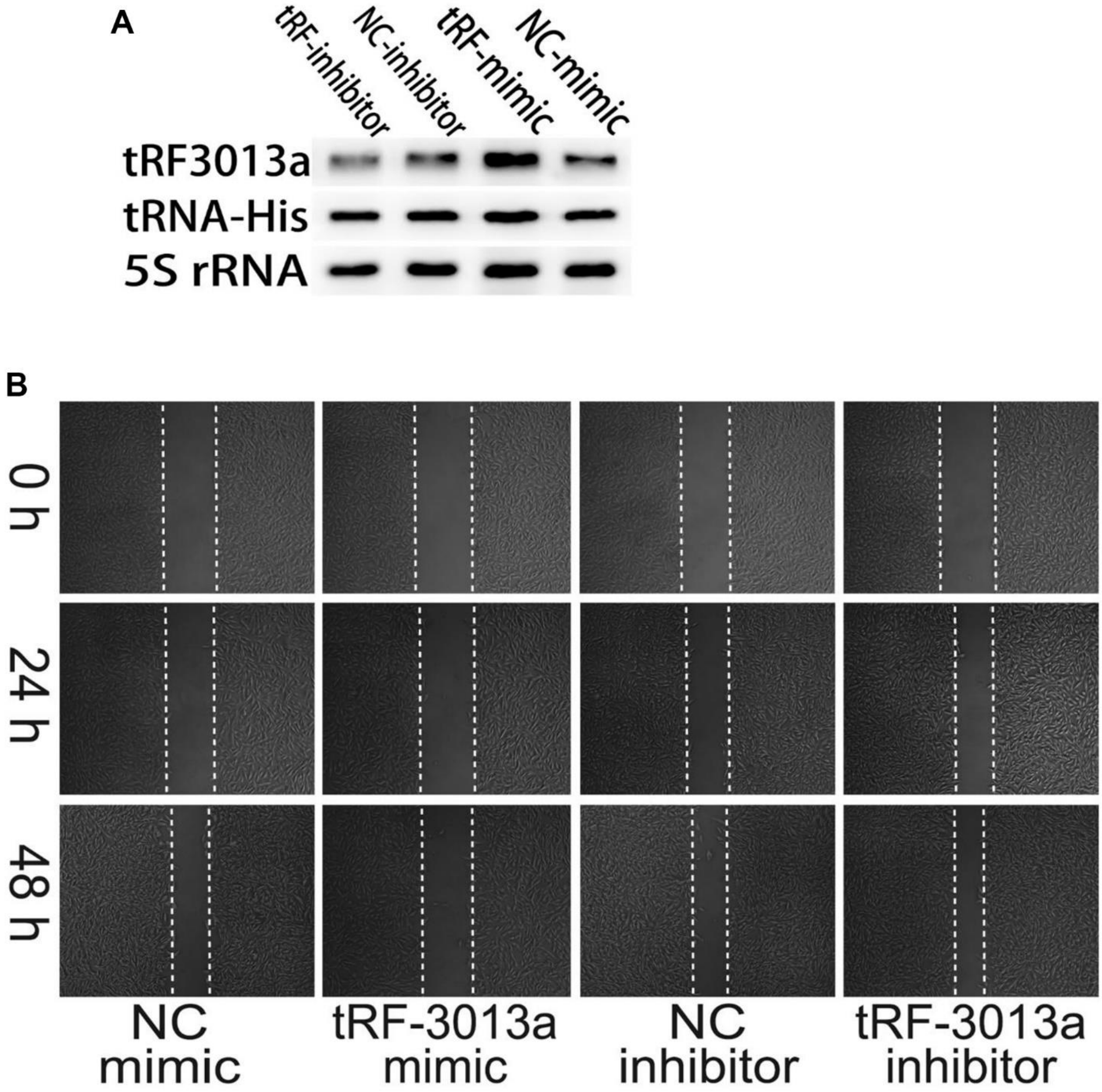

Supplementary Figure 5. (**A**) The Northern blotting analysis of tRFdb-3013a (tRF3013a, both P <0.05) and tRNA-His-GUG (tRNA-His) in the cells transfected with tRFdb-3013a mimic (tRF-mimic) or inhibitor (tRF-inhibitor) or negative controls (NC-mimic, NC-inhibitor). (**B**) Relative gap analysis scratch assay was used to assess the migration ability of SW480 cell.

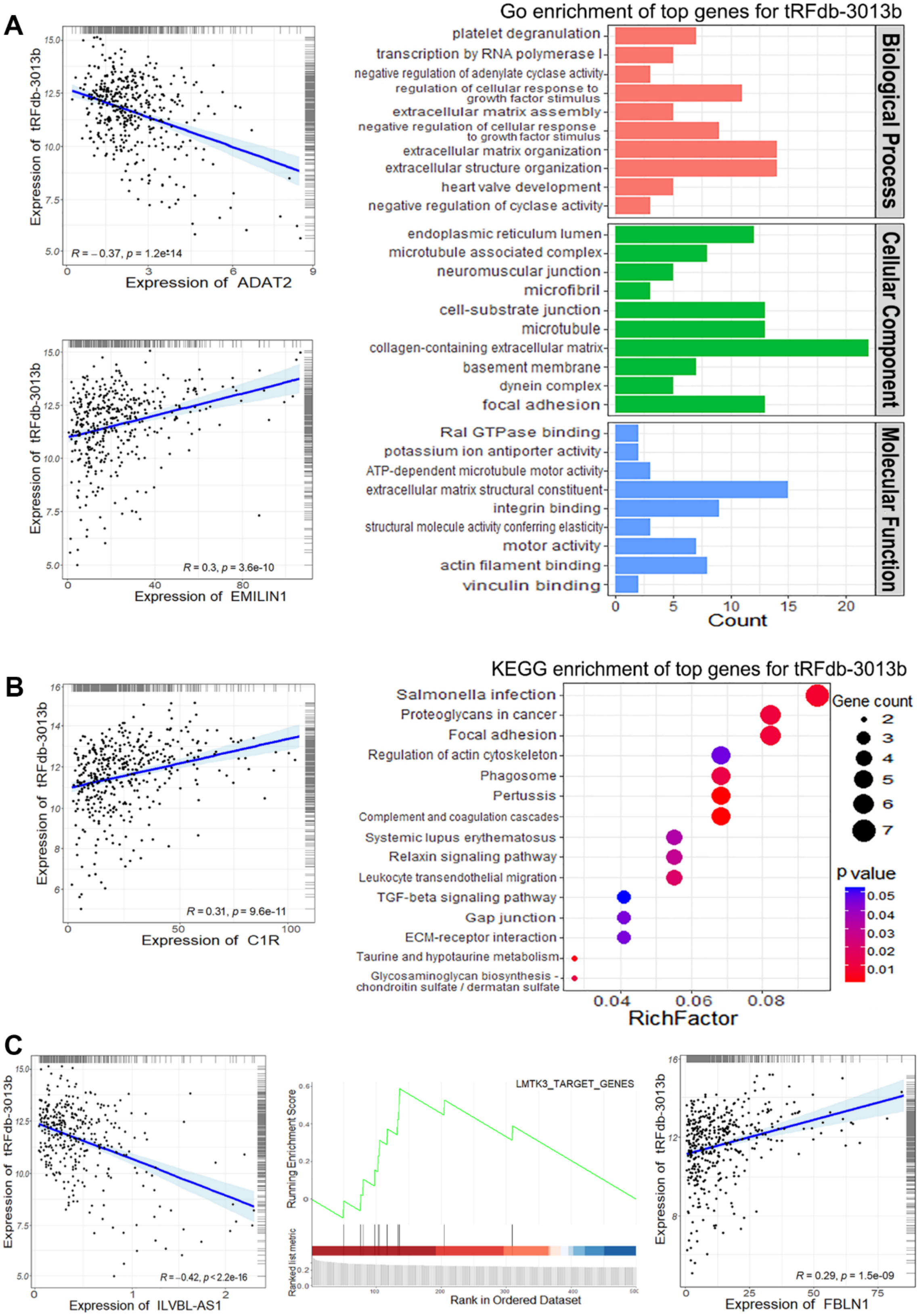

Supplementary Figure 6. The enrichment analyses of tRFdb-3013b correlated-genes within TCGA-COAD datasets. (**A**) The top gene ontology (GO) terms, including biological process, cellular component and molecular function. (**B**) The top KEGG pathway for tRFdb-3013b correlated-genes. The correlation scatter-plots of tRFdb-3013b and its correlated-genes (ADAT2, EMILIN1, and C1R). (**C**) GSEA (gene set enrichment analysis) plots of the molecular signatures, the scatter plots for tRFdb-3013b and its correlated-genes (ILVBL-AS1, FBLN1).

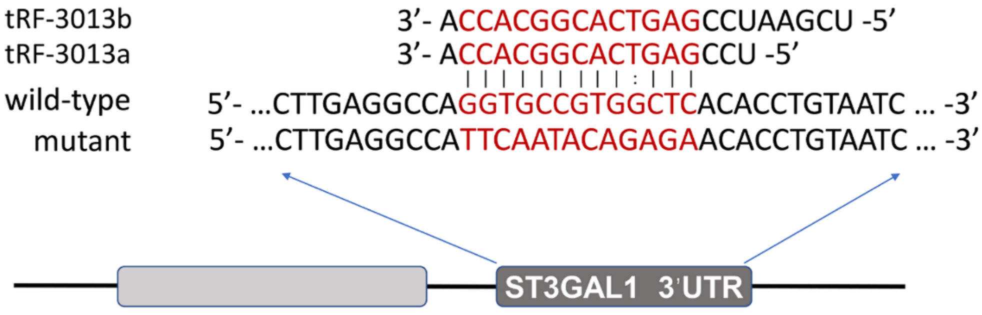

Supplementary Figure 7. Schematic diagram of the interaction position between tRFdb-3013a/b and the seed regions within the 3’UTR wide-type region and mutation region of ST3GAL1 gene in the luciferase reporter assay.

A

tDRnamer ID	Fragment sequence	length	MINTbase ID	reads	RPM
tDR-61:76-His-GTG-1-M2	**CCGAGTCACGGCACCA**	16	tRF-16-308HP1B	**541**	32.08
tDR-60:76-His-GTG-1-M2	**TCCGAGTCACGGCACCA**	17	tRF-17-8US5652	**947**	72.07
tDR-59:76-His-GTG-1-M2	A**TCCGAGTCACGGCACCA**	18	tRF-18-HS16MFD2	**416**	18.58
tDR-58:76-His-GTG-1-M2	AA**TCCGAGTCACGGCACCA**	19	tRF-19-DR5MEJE2	49	6.998
tDR-57:76-His-GTG-1-M2	GAA**TCCGAGTCACGGCACCA**	20	tRF-20-OML32826	61	17.99
tDR-56:76-His-GTG-1-M2	CGAA**TCCGAGTCACGGCACCA**	21	tRF-21-LE308HP1B	**321**	42.75
tDR-60:75-His-GTG-1-M2	**TCCGAGTCACGGCACC -**	16	tRF-16-8US565D	**162**	27.8
tDR-59:75-His-GTG-1-M2	A**TCCGAGTCACGGCACC -**	17	tRF-17-HS16MFJ	7	3.955
tDR-58:75-His-GTG-1-M2	AA**TCCGAGTCACGGCACC -**	18			
tDR-57:75-His-GTG-1-M2	GAA**TCCGAGTCACGGCACC -**	19	tRF-19-OML328FV	1	2.008
tDR-56:75-His-GTG-1-M2	CGAA**TCCGAGTCACGGCACC -**	20	tRF-20-LE308HP1	17	6.55
tDR-60:74-His-GTG-1-M2	**TCCGAGTCACGGCAC - -**	15			
tDR-59:74-His-GTG-1-M2	A**TCCGAGTCACGGCAC - -**	16	tRF-16-HS16MFD	5	4.889
tDR-58:74-His-GTG-1-M2	AA**TCCGAGTCACGGCAC - -**	17			
tDR-57:74-His-GTG-1-M2	GAA**TCCGAGTCACGGCAC - -**	18	tRF-18-OML328D1	1	1.176
tDR-56:74-His-GTG-1-M2	CGAA**TCCGAGTCACGGCAC - -**	19	tRF-19-LE308HI1	3	5.345
tDR-59:73-His-GTG-1-M2	**ATCCGAGTCACGGCA - - -**	15			
tDR-58:73-His-GTG-1-M2	AA**TCCGAGTCACGGCA- - -**	16			
tDR-57:73-His-GTG-1-M2	GAA**TCCGAGTCACGGCA- - -**	17	tRF-17-OML3282	1	1.225
tDR-56:73-His-GTG-1-M2	CGAA**TCCGAGTCACGGCA- - -**	18	tRF-18-LE308HDU	5	2.312

B

tDRnamer ID	Fragment sequence	length	MINTbase ID	reads	RPM
tDR-55:76-His-GTG-1-M2	**TCGAATCCGAGTCACGGCACCA**	22	tRF-22-WB8US5652	**6503**	62.09
tDR-54:76-His-GTG-1-M2	T**TCGAATCCGAGTCACGGCACCA**	23	tRF-23-YUHS16MFD2	**100**	35.37
tDR-53:76-His-GTG-1	GT**TCGAATCCGAGTCACGGCACCA**	24	tRF-24-7SDR5MEJE2	86	14.38
tDR-52:76-His-GTG-1	GGT**TCGAATCCGAGTCACGGCACCA**	25	tRF-25-R9OML32826	29	5.159
tDR-51:76-His-GTG-1	CGGT**TCGAATCCGAGTCACGGCACCA**	26	tRF-26-MNLE308HP1B	12	3.181
tDR-50:76-His-GTG-1	TCGGT**TCGAATCCGAGTCACGGCACCA**	27	tRF-28-48YUHS16MFD2	8	1.718
tDR-55:75-His-GTG-1-M2	**TCGAATCCGAGTCACGGCACC -**	21	tRF-21-WB8US565D	**1541**	16.82
tDR-54:75-His-GTG-1-M2	T**TCGAATCCGAGTCACGGCACC -**	22	tRF-22-YUHS16MFJ	15	2.22
tDR-53:75-His-GTG-1	GT**TCGAATCCGAGTCACGGCACC -**	23	tRF-23-7SDR5MEJV	4	1.443
tDR-52:75-His-GTG-1	GGT**TCGAATCCGAGTCACGGCACC -**	24			
tDR-51:75-His-GTG-1	CGGT**TCGAATCCGAGTCACGGCACC -**	25			
tDR-50:75-His-GTG-1	TCGGT**TCGAATCCGAGTCACGGCACC -**	26			
tDR-55:74-His-GTG-1-M2	**TCGAATCCGAGTCACGGCAC - -**	20	tRF-20-WB8US565	**113**	3.881
tDR-54:74-His-GTG-1-M2	T**TCGAATCCGAGTCACGGCAC - -**	21	tRF-21-YUHS16MFD	18	5.239
tDR-53:74-His-GTG-1	GT**TCGAATCCGAGTCACGGCAC - -**	22	tRF-22-7SDR5MEJ1	5	2.81
tDR-52:74-His-GTG-1	GGT**TCGAATCCGAGTCACGGCAC - -**	23			
tDR-51:74-His-GTG-1	CGGT**TCGAATCCGAGTCACGGCAC - -**	24	tRF-24-MNLE308HI1	1	1.298
tDR-50:74-His-GTG-1	TCGGT**TCGAATCCGAGTCACGGCAC - -**	25	tRF-26-48YUHS16MFD	5	18.6
tDR-55:73-His-GTG-1-M2	**TCGAATCCGAGTCACGGCA - - -**	19	tRF-19-WB8US5IU	44	5.828
tDR-54:73-His-GTG-1-M2	T**TCGAATCCGAGTCACGGCA - - -**	20	tRF-20-YUHS16MF	25	12.33
tDR-53:73-His-GTG-1	GT**TCGAATCCGAGTCACGGCA - - -**	21	tRF-21-7SDR5MEJB	14	10.81
tDR-52:73-His-GTG-1	GGT**TCGAATCCGAGTCACGGCA - - -**	22	tRF-22-R9OML3282	2	1.378
tDR-51:73-His-GTG-1	CGGT**TCGAATCCGAGTCACGGCA - - -**	23	tRF-23-MNLE308HDU	1	1.608
tDR-50:73-His-GTG-1	TCGGT**TCGAATCCGAGTCACGGCA - - -**	24	tRF-25-48YUHS16MF	2	1.075

Supplementary Figure 8. Representative images of the tRFdb-3013a (**A**) or tRFdb-3013b (**B**) sequence alignments of some fragments derived tRNA-His-GTG from tRF fragments within tDRnamer and MINTbase v2.0.
