# Supplementary material for: The tsRNAs (tRFdb-3013a/b) serve as novel biomarkers for colon adenocarcinomas
Source: Aging (Albany NY). 2024 Mar 6;16(5):4299–326. doi: 10.18632/aging.205590 (PMC10968714; doi:10.18632/aging.205590)
Supplement: Supplementary Table 2 [file aging-16-205590-s004.pdf]

Supplementary Table 2. Primers used in real-time PCR assay.

Primer names	Primer sequences (5´-3´)
ST3GAL1 forward	CACCCACACCCCTGTATTCTCC
ST3GAL1 reverse	TGTCCTGACCCAAGCTCAATGC
ACTB forward	CATGTACGTTGCTATCCAGGC
ACTB reverse	CTCCTTAATGTCACGCACGAT
tRFdb-3013a forward	TCCGAGTCACGGCACCA
tRFdb-3013b forward	TCGAATCCGAGTCACGGCACCA
U6 forward	GGAACGATACAGAGAAGATTAGC
U6 reverse	TGGAACGCTTCACGAATTTGCG
